# Global mpox lineage discovery and rapid outbreak tracking with nanopore sequencing

**DOI:** 10.1186/s12985-023-02059-2

**Published:** 2023-05-06

**Authors:** Michael S. Bosmeny, Adam A. White, Adrian A. Pater, Jennifer Crew, Joshua Geltz, Keith T. Gagnon

**Affiliations:** 1 Department of Biochemistry and Molecular Biology, Southern Illinois University School of Medicine, Carbondale, IL USA; 2 Department of Chemistry and Biochemistry, Southern Illinois University, Carbondale, IL USA; 3 Illinois Department of Public Health, Springfield, IL USA

## Abstract

Insufficient tracking of virus introduction, spread, and new lineage emergence for the human monkeypox (mpox) virus 1 (hMPXV1) outbreak of 2022 hindered epidemiological studies and public health response. hMPXV1 mutations accumulated unexpectedly faster than predicted. Thus, new variants with altered pathogenicity could emerge and spread without early detection. Whole genome sequencing addresses this gap when implemented but requires widely accessible and standardized methodologies to be effective both regionally and globally. Here we developed a rapid nanopore whole genome sequencing method complete with working protocols, from DNA extraction to phylogenetic analysis tools. Using this method, we sequenced 84 complete hMPXV1 genomes from Illinois, a Midwestern region of the United States, spanning the first few months of the outbreak. The resulting five-fold increase in hMPXV1 genomes from this region established two previously unnamed global lineages, several mutational profiles not seen elsewhere, multiple separate introductions of the virus into the region, and the likely emergence and spread of new lineages from within this region. These results demonstrate that a dearth of genomic sequencing of hMPXV1 slowed our understanding and response to the mpox outbreak. This accessible nanopore sequencing approach makes near real-time mpox tracking and rapid lineage discovery straightforward and creates a blueprint for how to deploy nanopore sequencing for genomic surveillance of diverse viruses and future outbreaks.

**Supplementary Information:**

The online version contains supplementary material available at 10.1186/s12985-023-02059-2.

## Introduction

Since its first identification in May 2022, the current outbreak of mpox virus, formerly the monkeypox virus [1], has been the largest observed in non-endemic countries. While there have been over eighty-five thousand mpox cases worldwide to date during this outbreak, less than 6,000 viral genomes have been sequenced and shared publicly [2, 3]. Additionally, the rate of single-nucleotide polymorphisms (SNPs) is higher than would be expected and novel deletions have been identified. General orthopoxviruses are estimated to acquire one or two substitutions per genome per year. However, the current mpox outbreak has over 50 SNP mutations more than the 2018/2019 reference strains [4]. In the months since the outbreak began, half a dozen more mutations have accumulated in some lineages. This equates to a clock rate of about 11–12 mutations per genome per year, several-fold more than expected. It is therefore conceivable that new variants with gain-of-function mutations or altered viral pathogenicity could arise.

**Fig. 1:**
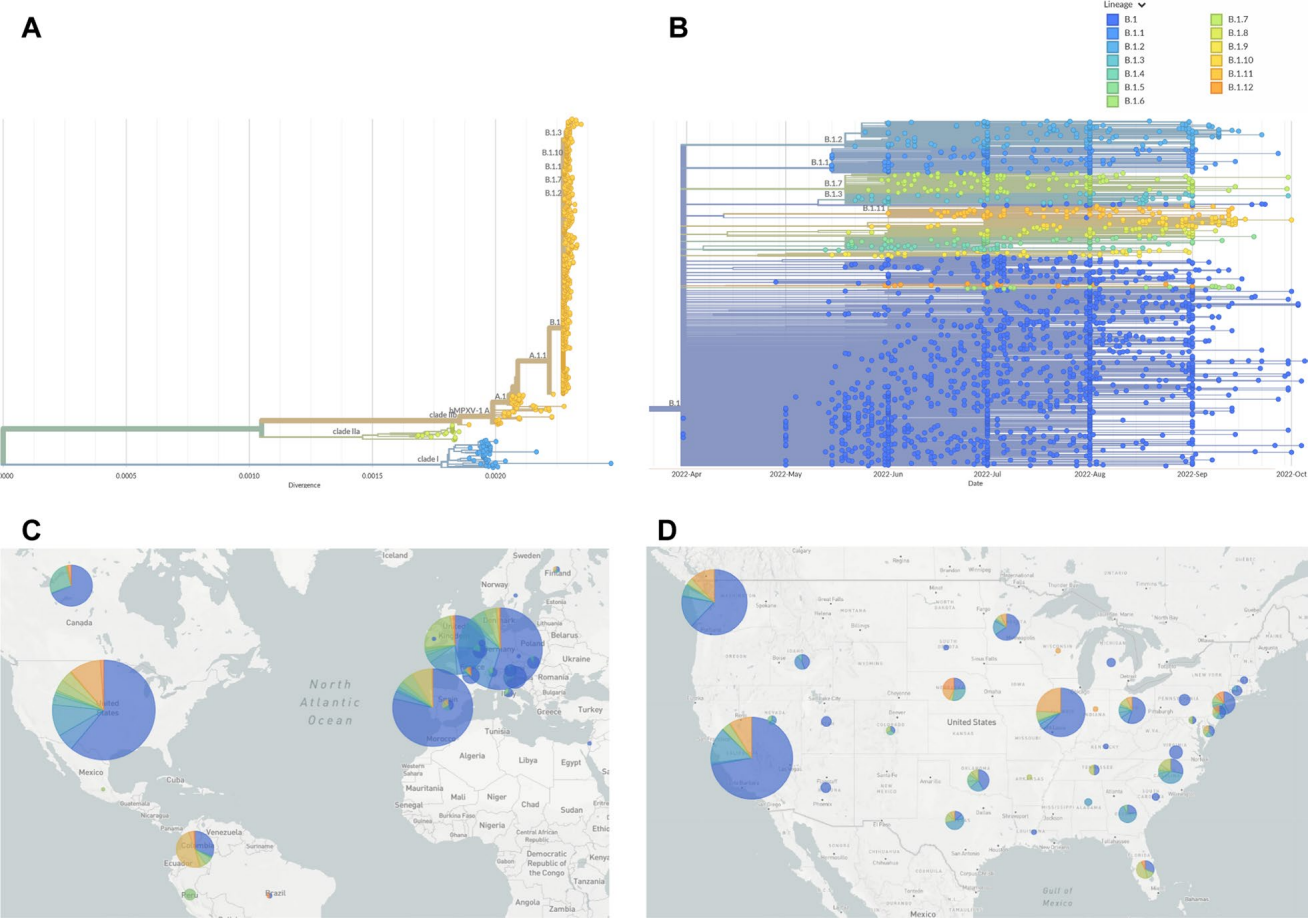
**A** Phylogenetic tree of sequenced Mpox cases, covering multiple clades. The current outbreak is Clade IIb. **B** Phylogenetic tree of only Clade IIb, lineage B.1 Mpox cases. Color coded based on lineages within B.1. **C** World map showing distribution of sequences and lineages. The larger the circle, the more sequences. **D** United States map showing distribution of sequences and lineages

Described in 1970 as an infection in humans distinct from other orthopoxviruses, mpox has similar symptoms to smallpox: fever, headaches, enlarged lymph nodes, and a rash leading to lesions [5]. These lesions, which cause the skin to crust, can also lead to secondary bacterial infections. Prior to the current outbreak, most human cases were observed in Africa, specifically West Africa, which were classified as Clade II, and the Congo Basin, which were classified as Clade I [6] (Fig. 1A). The current outbreak is an offshoot of the West African Clade II but has sufficient mutations to be categorized as Clade IIb, also known as human mpox virus 1 (hMPXV1). Within Clade IIb, there is a fine-scale classification based on a lineage naming scheme, for example A, A.1, A.1.1, and so forth. Nearly all the current outbreak sequences, by this categorization, fall into lineage B.1 (Fig. 1B). The current geographical and lineage makeup of mpox genome sequences in the world and United States are shown in Fig. 1C, D, respectively.

During the SARS-CoV-2 pandemic, there were publicly-available protocols to sequence viral genomes via large staggered amplicons using the Oxford Nanopore Technologies (ONT) MinION nanopore platform [7]. This allowed many smaller labs worldwide to sequence local samples quickly and without costly equipment. To facilitate a similar system for mpox, we developed an all-in-one MinION approach that retrofitted similar reagents and software that we and others previously used for SARS-CoV-2 genome sequencing [8]. This approach provides an accessible methodology that allows a greater number of laboratories to begin sequencing and submitting mpox genomes to global databases. It also serves as a blueprint for how to rapidly design, deploy, and share standardized and accessible genome sequencing technology for virtually any viral pathogen during regional or global outbreaks.

Our rapid targeted nanopore sequencing method was used to sequence 103 mpox patient samples from the state of Illinois, a Midwest region of the United States, resulting in 84 full genomes. Sample processing and sequencing of 1–24 samples could be accomplished in as little as 48 h by a single researcher. This sequencing revealed new insight into the regional introduction and emergence of lineages, as well as their subsequent spread, which included discovery of new lineages that may have emerged in the Midwest region and spread internationally. Thus, regional efforts by even small laboratories equipped with practical sequencing tools can have a large impact on the global scientific consensus for a disease epidemic like mpox. While the current mpox outbreak appears to be subdued, future outbreaks are possible. In addition, amplicon paneling with nanopore sequencing is a flexible platform that can be retrofitted to achieve rapid genomic surveillance for a diverse array of pathogenic viruses to help track and control outbreaks.

## Results

### Targeted amplicon paneling and nanopore sequencing

**Fig. 2:**
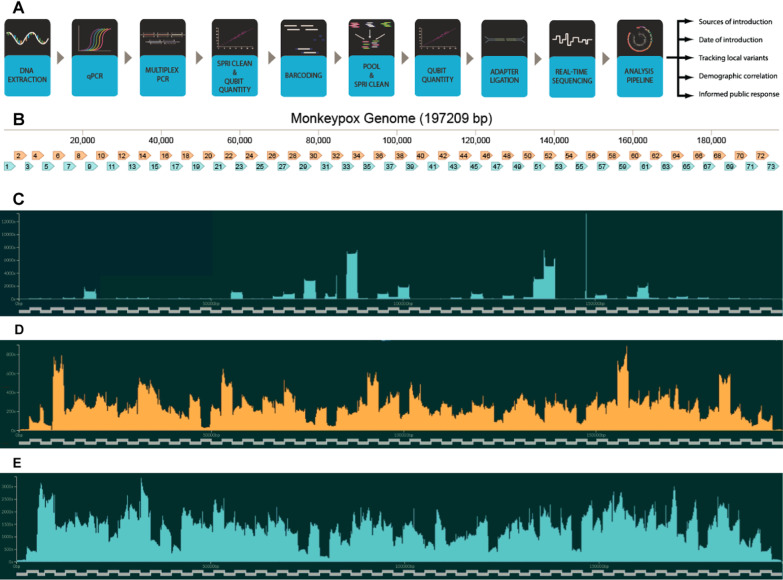
**A** Diagram showing workflow for preparation and sequencing of mpox sample. **B** Amplicon Map of Mpox Genome. Pool 1 amplicons are shown in blue, while Pool 2 amplicons are shown in orange. Amplicons 1 and 73 are inverted matches of each other, as are amplicons 2 and 72. **C–E** Coverage of individual amplicons in the Mpox genome during sequencing. Derived from FASTQ reads using RAMPART software [7]. The tiled amplicons are shown as the X-axis. The Y-axis indicates how many reads match that location in the genome. **C** First sequencing attempt of genome USA-2003, using USA-2003 as a reference genome. (**D**) Refined sequencing attempt of USA-2003. **E** An example sequencing attempt of a modern 2022 outbreak genome, using a lineage B.1 sequence as the reference genome

A set of primer pools, protocols, and computational commands were designed to sequence hMPXV1 genomes on a MinION nanopore flow cell (Fig. 2A). All necessary protocols are briefly described in the Methods section and available as Additional file 2: *Working Laboratory Protocols*. Initial amplicon primers were designed using PrimalScheme [9], which constructs a series of tiled amplicons of set size using a known genetic template, against NCBI reference sequence NC_063383.1, a Clade IIb, Lineage A sequence from 2018. To ensure that the primers had the maximum compatibility with both major clades of mpox and modern outbreak samples, six different genomes encompassing mpox samples from 2003 to the modern day were aligned and the designed primers compared against them. Only primers that matched all six genomes, indicating they resided in areas of low genetic variability, were retained. Those that contained mismatches were redesigned. These ensure the highest likelihood of the primers working with new mutations of mpox in this and future outbreaks. The final design incorporated 73 tiled amplicons of approximately 3,000 base-pairs (bp) in length, which were divided up into two pools. Pool 1 contained primers for odd-numbered amplicons and Pool 2 contained primers for even-numbered amplicons (Fig. 2B). Due to the terminal repeat regions (~ 6,400 bp) at the ends of the genome, known as the inverted terminal repeats (ITRs), amplicons 1 and 73 have the same primers, as do amplicons 2 and 72. Amplicons 3 and 71 have the same nested outer primers, but different internal primers.

PCR amplification with primer pools was tested against a known mpox virus genome, USA-2003 from a 2003 outbreak in Wisconsin, which was cultured in green monkey cells (BEI Resources, NR-4928). Because commercial samples contained low virus copy numbers, an unbiased whole-genome amplification method was first used prior to PCR amplification. Resulting amplicons from each primer pool after PCR were combined, barcoded, and sequenced on a MinION instrument. Reads were used to generate a consensus sequence. This initial PCR and sequencing attempt only yielded 20 × coverage of 78.3%, which limits full genome acquisition since tools like Nanopolish require 20 × coverage to call a consensus sequence. Amplicon distribution was skewed towards producing a high number of a handful of amplicons. In addition, only 52% of the total reads mapped to the mpox genome, indicating that non-specific amplification was occurring (Fig. 2C).

After careful analysis, primers prone to off-target amplification of green monkey or human genomic DNA were identified and replaced. We also found that amplicons 18, 20, 38, 40, 43, and 52 were slightly under-represented while amplicons 32 and 51 were over-represented. We subsequently doubled the concentration of primers for amplicons 18, 20, 38, 40, 43, and 52. Other changes included optimized thermocycler conditions and changing barcoding from a ligation-based method to a transposase-based rapid barcoding kit, which yielded better and faster results. A repeated sequencing attempt utilizing these changes had 20 × coverage of 97.10% and 97.42% of reads matched to the USA-2003 genome, indicating a clean PCR amplification (Fig. 2D). The resulting consensus sequence was found to exactly match the expected USA-2003 genome in all regions with sufficient coverage.

We then deployed this method to sequence current 2022 mpox-infected patient samples from the state of Illinois across the first few months of the outbreak. Magnetic bead-based DNA extraction was performed, followed by viral load quantification by quantitative PCR (qPCR). Testing facilities where samples were obtained used a generic non-variola *orthopoxvirus* (NVO) qPCR probe designed by the Center for Disease Prevention and Control (CDC) for testing for the presence of mpox viral DNA. Other testing facilities may use mpox-specific CDC probe-based qPCR. This probe, while more precise in detecting mpox, has failed on a small subset of mpox samples first observed in California. This is due to partial ITR deletions where the qPCR probe binds. These samples were only identified by testing with the NVO qPCR. To guard against similar problems, we designed our own probe-based qPCR reaction, OPG123, based on analysis of low-mutational areas of the mpox genome. To standardize reads, sample DNA was tested in the same qPCR conditions and using the same baseline threshold value. In general, our cycle threshold (*C*_**t**_) values were approximately four *C*_**t**_ values higher than those generated by testing facilities (Additional file 1: Fig. S1). This could be due to different qPCR conditions, degradation during shipping, or freeze–thaw cycles.

To quantify the number of genomes needed for successful sequencing, a short double-stranded DNA fragment was synthesized and used for amplification with a known copy number (Additional file 1: Fig. S2). Based on these results, between 5,000 and 10,000 copies of the Mpox genome are needed for optimal amplicon production and sufficient genome sequence coverage. This correlated to a *C*_t_ value of 32 or lower with our qPCR probe OPG123. However, based on qPCR comparisons between our probe, the CDC mpox-specific probe, and an RNase P probe (human genomic DNA), this copy number limitation may be linked to the amount of mpox DNA compared to contaminating human DNA (Additional file 1: Fig. S3). Samples with low OPG123 *C*_**t**_ values also had a high ratio of Mpox DNA to human DNA (8–12 *C*_**t**_ difference) while samples with high OPG123 *C*_**t**_ values had lower ratios of Mpox DNA to human DNA (3–4 *C*_**t**_ difference).

After qPCR quantification, hMPXV1 genomic DNA was PCR amplified separately with primer Pool 1 and Pool 2. Resultant amplicons from the two reactions were then combined and proceeded through a series of clean up and library preparation steps (see Additional file 2: *Working Protocols*). After nanopore sequencing, which could be achieved by barcoding up to 24 samples on one flow cell, consensus sequences were generated for each sample. In total, 103 patient samples were processed, resulting in 84 full genomes based on consensus sequences. Additional file 1: Table S1 shows *C*_t_ values and 100x/20 × coverage numbers for these 84 samples. The mean coverage of the consensus sequences was 98.79%, with a median coverage of 99.41%. An example of 99.95% sequence coverage as viewed in Rampart is shown in Fig. 2E. One challenge to genome coverage was the terminal ITR repeats. Minimap2, the program used to align reads to a template genome, attempts to match these reads to one end of the genome or the other, but not both. This is an issue if there is a low number of reads in that region. This can result in uncalled bases (Ns) in Amplicons 1 and 73 in the final consensus. If insufficient reads are generated, one strategy is to remove the terminal repeat region from one end of the template genome so that minimap2 aligns all reads on the other terminal repeat. The removed repeat can then be manually re-added.

### A diverse set of viral introductions

After sequencing, all consensus sequences were analyzed using NextClade software [10]. This compares the sequence of interest against a generic Clade IIb, lineage B.1 template genome, based on ON563414.3, MPXV_USA_2022_MA001, a sample from the current outbreak collected in May 2022 in Massachusetts, USA, and highlights any indels or mutations. The sequences can then be categorized into specific sub-lineages within B.1. As of October 2022, there are twelve sub-lineages, as defined by the NextClade group [11] (Fig. 1B). Each has one to four unique nucleotide mutations. There are also the remaining approximately 60% of the current outbreak sequences that fall within the B.1 lineage, but do not fit within a designated sub-lineage. Our sequencing suggests that Illinois has seen a significant number of different viral introductions. Table 1 shows some of the mutations encountered in our sequencing.

**Table 1:** List of most common mutations found in sequenced mpox samples, as compared to a Clade IIb, Lineage B.1 template genome

Nucleotide mutations	Lineage name	Mutational pattern sequenced	Mutational pattern found elsewhere	Total
G186165A (OPG210:D1604N)	B.1.2	2	192	194
G74360A (OPG094:R194H)	B.1.1	3	189	192
C25644T	B.1.7	5	148	153
C18133T, G67611A, G130231A, and G159277A (OPG185:E121K)	B.1.11	21	68	89
G34308A (OPG053:S128L)	B.1.4	1	49	50
C132520T, G175093A (OPG204:D188N)	B.1.13	18	19	37
C77462T (OPG098:S188F)	–	4	21	25
G22814A (OPG038:H116Y), G55162A (OPG074:S655L), G137128A, G167910A (OPG193:E193K)	–	2	1	3
C88308T	–	3	0	3
C156533T	–	3	0	3

Resulting protein changes are in parentheticals after nucleotide changes

In addition, another eight sequences possessed unique mutations not in common with any of the other sequences in the NCBI database. These diverse mutational patterns in a small number of cases indicate that a substantial number of viral versions entered the region and spread.

### Novel B.1 lineage discovery

**Fig. 3:**
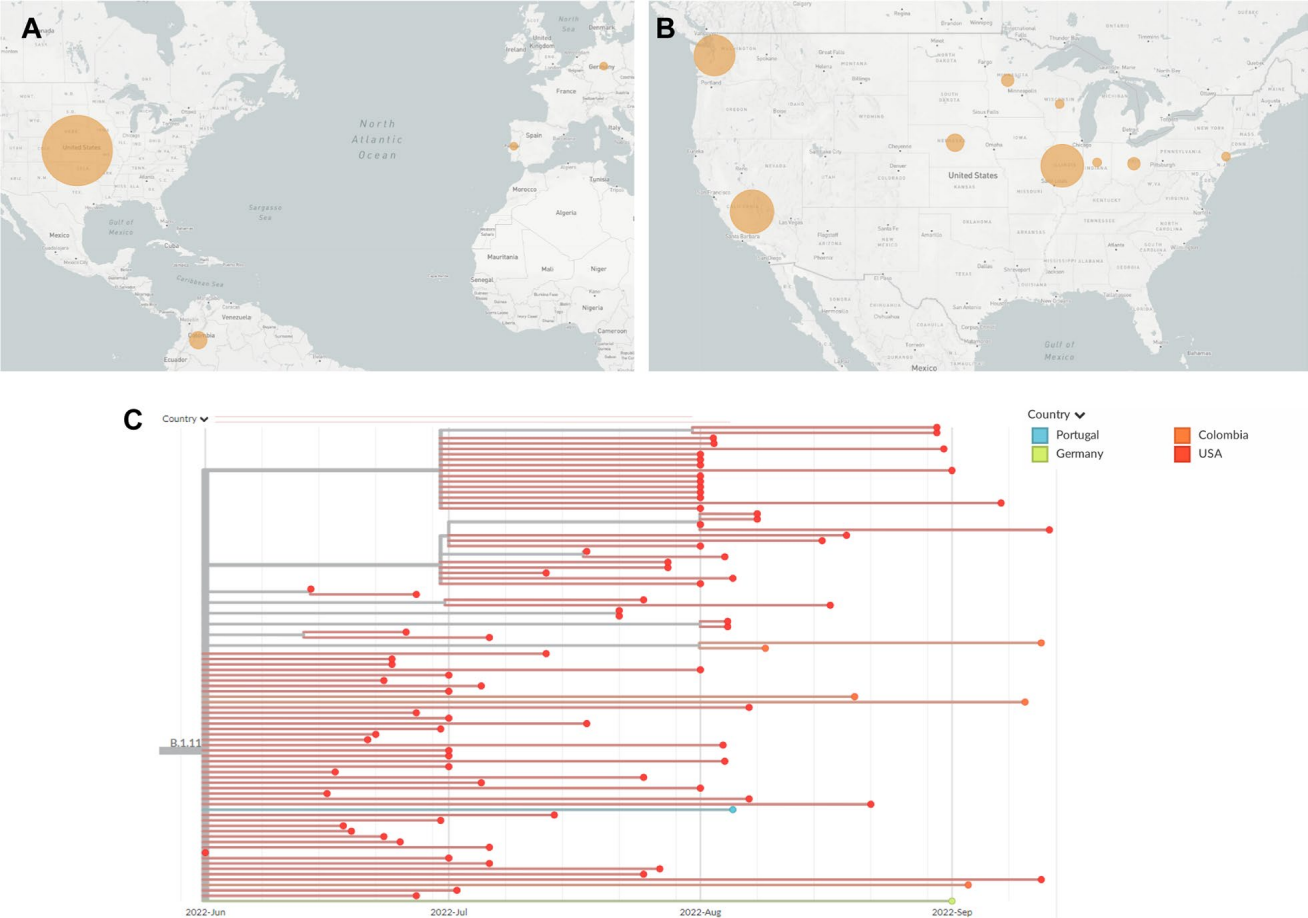
Geographic and phylogenetic data for Mpox lineage B.1.11. **A** World map showing lineage distribution by country. The larger the circle, the higher the number of sequences located there. Compare with total sequences in Fig. 1C. **B** United States map showing lineage distribution by state. Compare with total sequences in Fig. 1D. **C** Phylogenetic tree showing collection dates and locations for each sequence of B.1.11

Our genome sequencing also suggests that several B.1 lineages may have emerged in Illinois or the Midwest region. Of the 84 samples sequenced, a quarter of them had four mutations in common: C18133T, G67611A, G130231A, and G159277A (Fig. 3). Three of these mutations are in non-coding regions, while the last, G159277A, produces mutation E121K in protein OPG185. At the time of this sequencing, this mutation categorization was still unnamed. It has subsequently been designated as lineage B.1.11 by Nextclade. Currently, B.1.11 is the fifth-most common lineage in the current Mpox outbreak, with eighty-four total sequences. Our sequencing contributed 25% of the genomes needed to help establish this recently named lineage. This lineage is primarily found in the U.S., with just a few samples from outside North America: five August and September infections from Columbia, an early August infection in Portugal, and a September infection in Germany. Although the dearth of sequencing makes it difficult to know with certainty the origin of this lineage, the earliest cases are all found in Illinois in late June. It is not until July that similar strains appear in California, Washington, and other regions of the U.S.

**Fig. 4:**
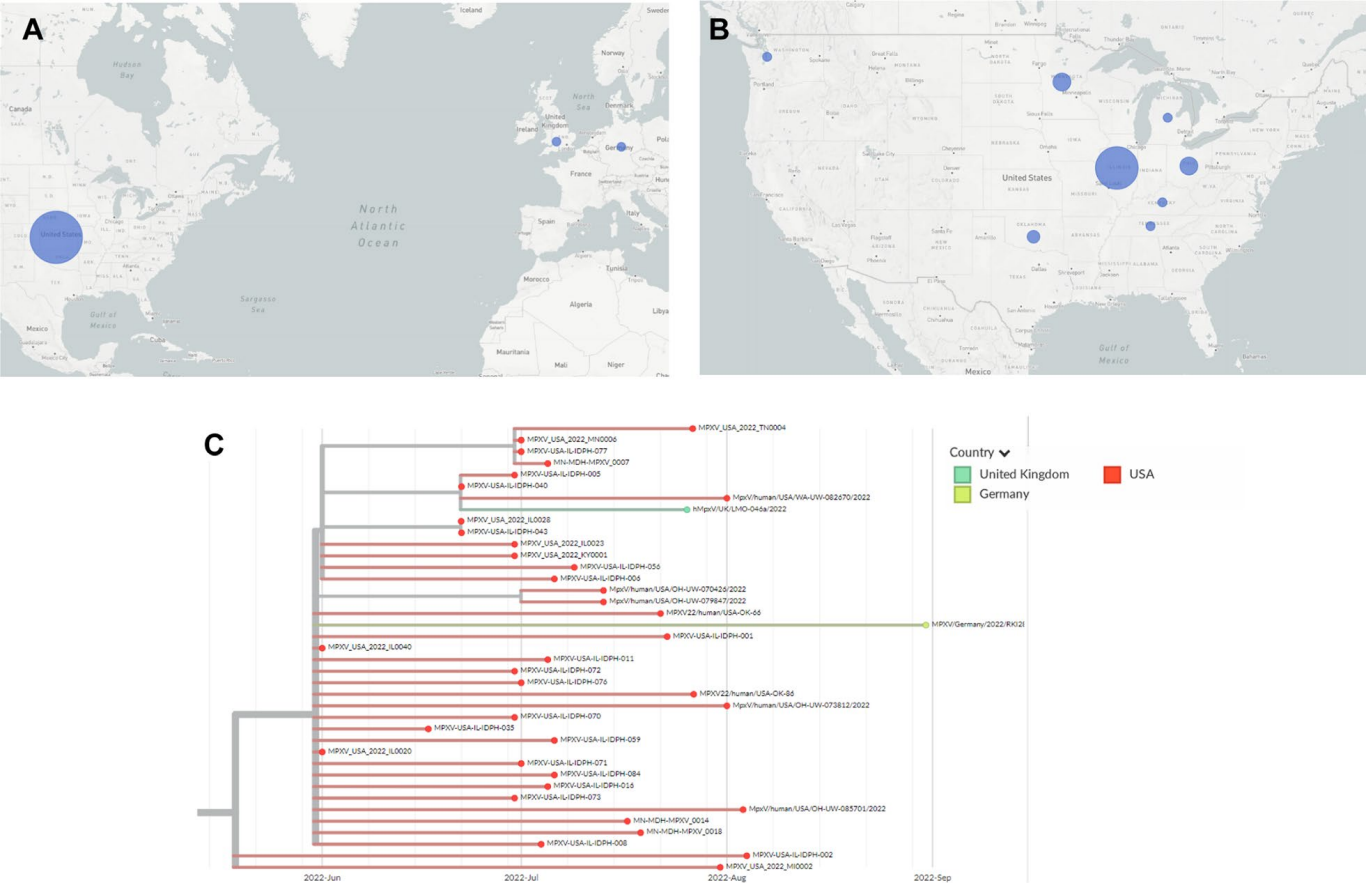
Geographic and phylogenetic data for Mpox lineage B.1.13. **A** World map showing lineage distribution by country. The larger the circle, the higher the number of sequences located there. Compare with total sequences in Fig. 1C. **B** United States map showing lineage distribution by state. Compare with total sequences in Fig. 1D. **C** Phylogenetic tree showing collection dates and locations for each sequence in B.1.13

Another major mutational pattern seen in our sequencing has only just recently received a lineage designation, B.1.13. (Fig. 4). Nineteen of our eighty-four samples have G175093A, and of those, eighteen have a secondary mutation of C132520T. G175093A confers the mutation D188N in protein OPG204, while C132520T is in a noncoding region. B.1.13 is primarily found in the Midwest of the U.S. It appears to have originated there, with infections appearing with these mutations in mid-June. From there, it appears to have gradually spread to other Midwestern areas, like Oklahoma, Minnesota, Ohio, Tennessee, and Kentucky. So far, no sequences with this mutational pattern have appeared in California and only a small number in Washington state, despite their high sequencing rate. Finally, in late July a similar sequence was seen in the United Kingdom and in late August another sequence in the group was seen in Germany.

Currently, B.1.13 is the eleventh most common lineage in the 2022 global outbreak, with a total of thirty-seven sequences. Half of the sequences in B.1.13 were generated from this study. This demonstrates the need for deeper regional sequencing and the ability of a small number of regional genomes to impact global epidemiology.

## Discussion

**Fig. 5:**
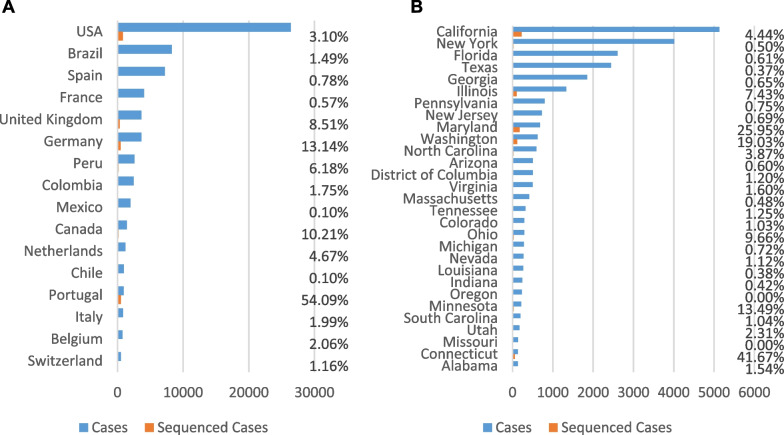
Comparisons of mpox cases versus mpox sequences for the current 2022 outbreak. **A** Countries with more than 500 cases. **B** US States with more than 100 cases

It seems likely that B.1.11 and B.1.13 lineages originated in the U.S. Midwest, possibly even Illinois given that Chicago is a travel hub. However, it is impossible to know with any certainty due to the lack of systematic mpox genome sequencing in the U.S. and internationally. Many U.S. states have less than a dozen sequences submitted. Washington, Connecticut, and California are standouts with high numbers of genome sequences, but they are exceptions. Our recent sequencing increased the number of genomes from Illinois by five-fold. There could certainly be new mutational variants appearing that have remained uncharacterized because sequencing is absent in many U.S. states, such as New York, Florida, and Texas, and very low in most other countries (Fig. 5). In Europe, both Portugal and Germany are submitting an impressive number of sequences. In contrast, Spain and France have uploaded samples for less than 1% of their total cases.

The 2022 mpox outbreak is noted to have 6–12 fold more mutations from previous 2018–2019 strains than expected, based on the calculated substitution rates for *orthopoxviruses* [4]. As a great number of these mutations are of the same pattern, GA → AA or TC → TT, it has been speculated that this new mutation rate may be driven by the human APOBEC3 enzyme. APOBEC is a family of cytidine deaminases that can attempt to disrupt viral replication by nucleotide alteration based on specific motifs within the nucleotides [12, 13]. APOBEC3 specifically targets TC positions and modifies the C to U, which, upon replication will cause TC to become TT. GA → AA mutations are the result of a TC → TT mutation on the other strand.

With limited genome sequencing and faster than expected mutation rates, it is likely that the full picture of pathogen evolution and host adaptation is not being captured. Although the number of mpox cases may wax and wane, the hMPXV1 virus may become endemic to many new international regions, including the U.S. While the likelihood seems low, individual mutations or groups of mutations could nevertheless arise that significantly alter virus pathogenicity, severity, or infectivity. With SARS-CoV-2, thanks to global sequencing efforts, public health officials are now quickly and regularly alerted to new, more contagious variants, like Omicron. A similar approach should be preemptively applied to mpox and all new viral outbreaks.

The hMPXV1 genome is six times larger than SARS-CoV-2 and possesses inverted terminal repeats, a number of microsatellites, and homopolymeric mononucleotide repeats, which all introduce sequencing challenges [14]. Nonetheless, genome sequencing shortfalls are not the result of these technical challenges but instead a lack of accessible resources. Most mpox virus genome sequencing has been performed with metagenomics shotgun approaches, which sequence everything and can be time consuming and expensive compared to targeted sequencing [15]. Near the end of this study, a commercially available kit was released for targeted sequencing on Illumina platforms. However, it is designed primarily for sequencing core facilities and private contract laboratories that may process large numbers of samples. In contrast, many small research groups and developing regions purchased and used nanopore sequencing supplies to sequence SARS-CoV-2 genomes during the COVID-19 pandemic. Thus, our approach should be nearly “plug-and-play” with existing resources for many research groups.

Amplicon paneling is a method introduced previously and developed by the ARTIC group for targeted SARS-CoV-2 sequencing [16]. It is the method used for Illumina and nanopore sequencing platforms. For nanopore sequencing, amplicon size is very flexible and can range from only a few hundred base-pairs up to tens of thousands of base-pairs. In contrast, Illumina technology has traditionally been severely limited to short reads, which requires substantially more amplicons to be generated. Larger amplicon sizes are particularly valuable for mpox genome sequencing due to repeat regions. For example, there is an ATATACATT motif that repeats dozens of times, starting at base 197,077. Small, 100–200 bp fragments that an Illumina sequencing run will generate may not fully span this repeat motif, and it is therefore extremely difficult to accurately count how many times the motif occurs. A look at current sequences (Additional file 1: Fig. S4) serves to illustrate this as over half of all sequences list a deletion in this area. Interestingly, there are no patterns to these deletions on the phylogenetic tree, leading to the conclusion that these deletions are sequencing artifacts. Nanopore sequencing does not have this problem because the amplicon can be larger than the repeat region, making it possible to know with certainty whether deletions actually exist. One disadvantage of nanopore sequencing is base calling for mononucleotide, or homopolymeric, repeats. Nanopore technology reads bases as grouped k-mers using a voltage-measurement as the nucleic acid strand passes through the pore. Long stretches of mononucleotides can sometimes be miscounted. Consensus-finding programs can help correct for this and newer flow cell chemistries can boast a higher base-calling accuracy.

In summary, we have developed a rapid nanopore sequencing method that should prove highly accessible to small laboratories and international groups participating in local mpox genome sequencing efforts. It borrows from the success of nanopore and amplicon tiling methods, can be high-throughput with 96-well plates and barcoding, and we provide full laboratory *Working Protocols* to support immediate implementation. The amplicon tiling primer sets created in this study are compatible with all known mpox genomes and were designed to extend to likely future variants. By employing this method on 103 regional samples, we sequenced 84 complete hMPXV1 genomes, which significantly increased the number of genome sequences regionally and nationally. It also offered unexpected insight into both regional and global dynamics of the current mpox outbreak. Multiple introductions of mpox occurred in the U.S. state of Illinois early in the outbreak, some of which then spread regionally. Well-placed travel restrictions or other public health policies might have limited the impact of the outbreak. In addition, multiple mutational profiles were observed in Illinois that have not been found elsewhere. It is possible that these emerged in Illinois or neighboring Midwest regions. New lineages of hMPXV1 were identified, one of which has been designated as B.1.11 on the global phylogenetic tree and one of which remains undesignated. Both of these lineages have a high likelihood of emergence within Illinois or the U.S. Midwest region. Our sequencing was able to fill gaps in the global phylogenetic tree that provide a better picture of the evolution of the virus and its adaptation to the human host. Finally, our approach creates a straightforward blueprint for adapting amplicon paneling and nanopore sequencing for accessible and affordable genomic surveillance to track nearly any viral outbreak where the virus’ genome sequence, or a close relative, is known. This blueprint should also be applicable to future mpox outbreaks.

## Methods

### Extraction of viral genomes

Initial testing and optimization was done with a genome acquired from BEI Resources (https://www.beiresources.org/Catalog/BEINucleicAcids/NR-4928.aspx). Low copy number in this sample was augmented with whole-genome amplification via Repli-G treatment (Qiagen, # 150023). All sequenced samples were obtained from the Illinois Department of Public Health.

A detailed protocol is available in Additional file 2: Working Protocol 1. Briefly, samples are stored at − 80 °C until ready for processing. Virus sample should be delivered in an inactivation (lysis) solution, referred to here as the viral transport media (VTM). Extractions should be done under sterile conditions in a BSL-2–class biosafety cabinet. For consistency, extraction and all future steps should also be performed on a negative control sample containing only VTM or phosphate-buffered saline (PBS). Any genomic sequences that show up in the negative control at the end of the workflow should be considered to be possibly present as a contamination in any other samples processed at the same time. DNA is extracted from virus samples using a magnetic bead-based process. First, DNA is bound to the magnetic bead in an isopropanol solution. After binding, a magnet is used to separate the beads and bound DNA from the supernatant liquid. The beads and DNA is then washed twice with an 80% ethanol solution. Finally, the DNA is eluted from the beads by the addition of a DNA elution buffer.

### qPCR to estimate viral load

To quantify the relative amount of viral genomic DNA in each extraction, qPCR is performed on each sample. In initial testing, we quantified DNA using two different probes: One which uses the same probe sequences as the CDC’s Mpox qPCR tests (Sequences: Forward: 5'-GGAAAATGTAAAGACAACGAATACAG-3', Reverse: 5'-GCTATCACATAATCTGGAAGCGTA-3', Probe: 5'-AAGCCGTAATCTATGTTGTCTATCGTGTCC-3') and one of our own design, called OPG123 (Sequences: Forward: 5'-GGGATGCTAATATTCCCAAACATAC-3’, Reverse: 5'-TCTAAATCCGCATTAGACACCTT-3', Probe: 5'-AGAGATATCAGCCGCAATAGCATCCC-3’). Later, we tested exclusively with OPG123. Both probes were manufactured by IDT. Typically, the standard manufacturer’s qPCR protocol for the PrimeTime gene expression assay was followed (Additional file 2: Working Protocol 2). Supplementary Fig. 5 shows negative and positive bands for these qPCR tests.

### Multiplex amplicon tiling PCR to amplify viral genomes

For all samples that have sufficient material to be sequenced, based on the qPCR results, the next step is to amplify the DNA using a series of 73 overlapping, 3000 base pair amplicons. To prevent interference between adjacent amplicons, odd-numbered amplicons are used in one pool, and even-numbered amplicons are used in a second pool. This is modeled after the ARTIC Network’s SARS-CoV-2 V3 sequencing primers, which produce a similar pattern of amplicons. NEB Q5 Hot-Start Polymerase was used for our PCR reactions (Additional file 2: Working Protocol 3). Several adjustments were made to the concentrations of the oligos to produce the best sequencing results:

It is recommended, at least for initial sequencing runs, to verify PCR products for each sample and pool on a 1% agarose gel via electrophoresis. Correct band sizes are approximately 3000 bp. Supplementary Fig. 6 shows an example of several samples checked on a gel, along with a negative control.

### Pooling, purification, and quantification of PCR amplicons

After PCR amplification, Pool 1 and Pool 2 for each virus sample are pooled together and cleaned of PCR reactants using SPRI AMPure XP beads (Additional file 2: Working Protocol 4). The ratio of bead solution to DNA solution can be varied to select out smaller DNA fragments, if desired. Initial testing, using 1:1 bead solution to DNA solution ratios, showed that there were many small, < 200 bp DNA fragments that occupied bandwidth in the later MinION sequencing reaction. By reducing the ratio down to 0.75:1 bead solution to DNA solution, an abundant amount of 3000 bp fragments were preserved while excluding many of these smaller products, giving a cleaner sequencing run.

After purification, the quantity of DNA in each sample needs to be determined. DNA was quantified using a Qubit 2.0 fluorometer and the Qubit double-stranded DNA assay kit (Additional file 2: Working Protocol 5). This quantification allows for an equal amount of each sample to be carried forward to future steps and, ideally, an equal number of reads per sample when the finished process is loaded into the flow cell for sequencing.

### DNA library barcoding and adapter ligation

To prepare the amplicons for sequencing, our lab utilized the Oxford Nanopore RAPID Barcoding kit. This kit uses a transposase which cleaves template and attaches barcoded tags to the cleaved ends (Additional file 2: Working Protocol 6). Afterwards, the barcoded samples were pooled, and then cleaned via bead extraction. Rapid adapters were added to pooled barcoded samples and allowed to attach by a room temperature incubation (Additional file 2: Working Protocol 7).

### Nanopore sequencing with a MinION

Sequencing was done using the Oxford Nanopore Technologies MinION Mk1B (MIN-101B) sequencer and Spot-ON Flow Cell, R9 version (FLO-MIN106D). Oxford Nanopore’s MinKNOW software (v21.11.9) was used to read from the flow cell and base-call the reads (Additional file 2: Working Protocol 8).

During sequencing, ARTIC’s RAMPART software (v1.0.6) was used to monitor the reads on each barcode separately and the respective coverage of the Mpox genome (Additional file 2: Working Protocol 9).

### Phylogenetic analysis, variant calling, and database depositing

The ARTIC bioinformatics pipeline (v1.2.1) was used to consolidate FASTQ reads from MinKNOW and align them against the NextClade B.1 Mpox template genome (Additional file 2: Working Protocol 10).

NextClade software was used to identify mutations in each sequence against the template genome (Additional file 2: Working Protocol 11).

All sequences were uploaded into the NCBI GenBank system using their BankIt upload process.

## Supplementary Information

**Additional file 1.** Supplementary Figures and Table.

**Additional file 2.** WorkingProtocols.

## Data Availability

All sequencing data is available. Complete consensus mpox genome sequences have been deposited at the National Center for Biotechnology Information (NCBI). Accession numbers correlating with each sample are listed in Additional file 1: Table S1. Compressed fastq files for all sequencing runs are available at https://zenodo.org/record/7659572. Fast5 files of raw nanopore data for all sequencing runs are available upon request due to the large file size associated with the fast5 file type.
