# Supplementary material for: Global mpox lineage discovery and rapid outbreak tracking with nanopore sequencing
Source: Virol J. 2023 May 6;20:90. doi: 10.1186/s12985-023-02059-2 (PMC10163848; doi:10.1186/s12985-023-02059-2)
Supplement: Supplementary file 1 — Additional file 1. Supplementary Figures and Table. [file 12985_2023_2059_MOESM1_ESM.pdf]

Supplementary Data

Global Mpox Lineage Discovery and Rapid Outbreak Tracking with Nanopore Sequencing

Michael S. Bosmeny[1], Adam A. White[1,†], Adrian A. Pater[2,†], Jennifer Crew[3], Joshua Geltz[3], and Keith T. Gagnon[1,2,*]

[1] Department of Biochemistry and Molecular Biology, Southern Illinois University School of Medicine, Carbondale, IL, USA.
[2] Department of Chemistry and Biochemistry, Southern Illinois University, Carbondale, IL, USA.
[3] Illinois Department of Public Health, Springfield, IL, USA.
[†] These authors contributed equally.
* Corresponding Author: ktgagnon@siu.edu

Supplementary Figures

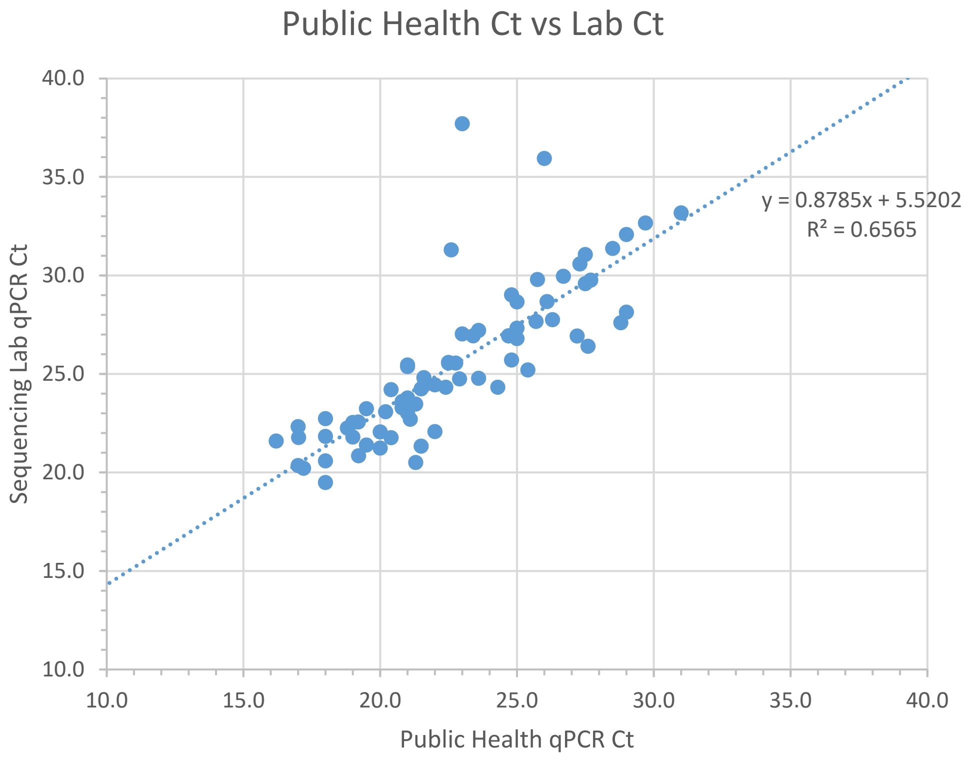

Supplementary Figure S1: Comparison of qPCR Ct values from Illinois Department of Public Health and those generated using OPG123 qPCR probes.

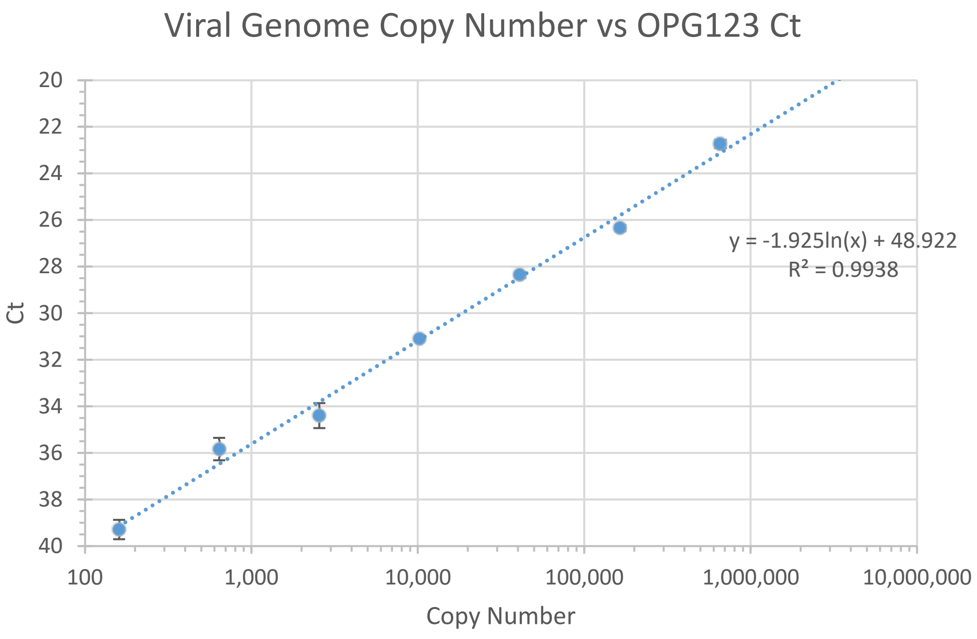

Supplementary Figure S2: Comparison of number of copies of a DNA gBlock and the qPCR Ct values generated from them using the OPG123 qPCR probe set.

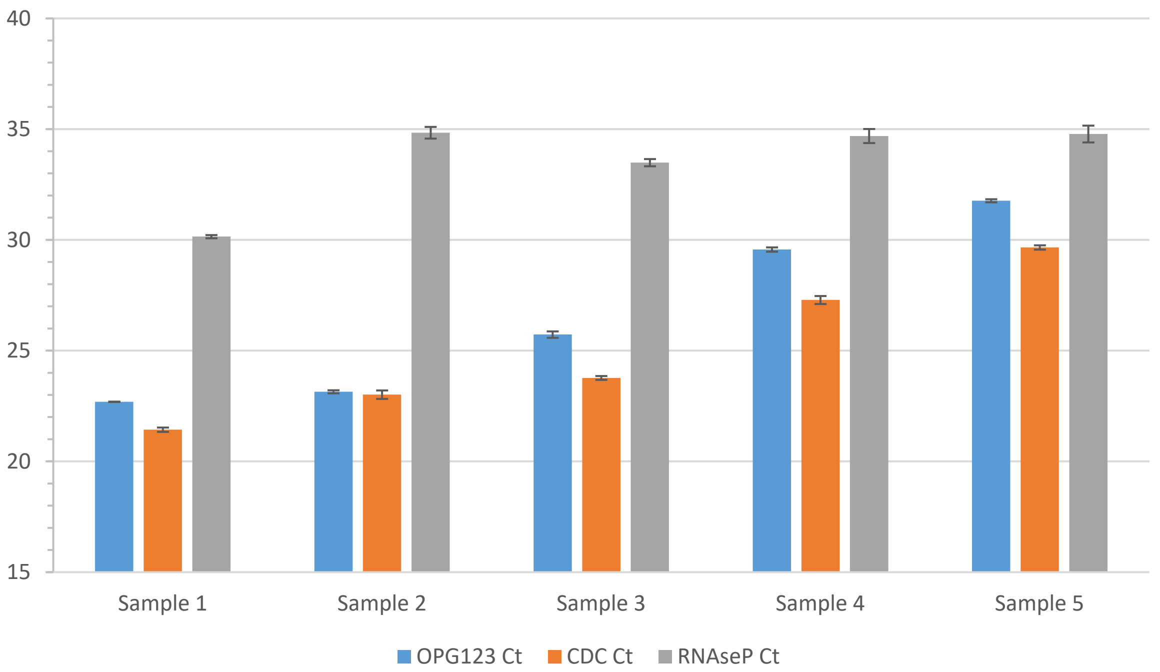

Supplementary Figure S3: Ct values for five different mpox samples, using three different qPCR probes. Each sample/probe combination was replicated three times.

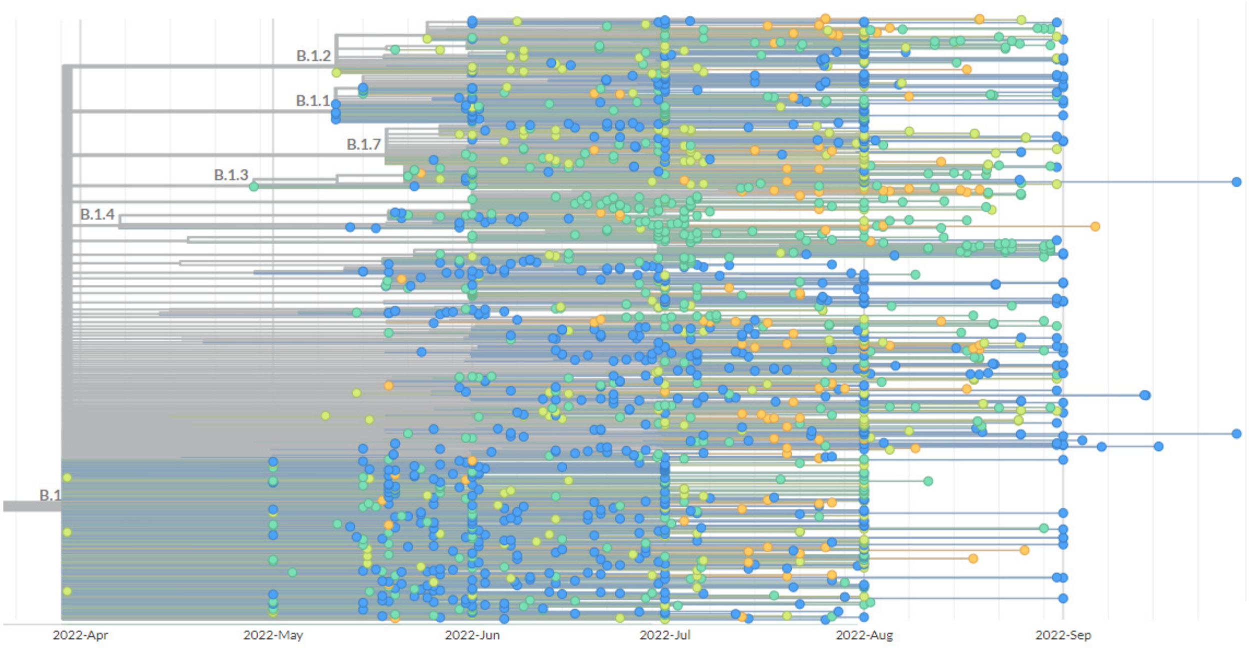

Supplementary Figure S4: Phylogenetic tree showing Clade IIb, Lineage B.1 mpox sequences. Color of each sequence indicates status of indicated deletion starting at base 179077. Teal: no deletion. Lime-green: Deletion to base 179094 (17 bases removed). Blue: Deletion to base 179148 (71 bases removed). Orange: Deletion to base 179175 (98 bases removed). Each lineage follows no clear pattern in deletions, indicating this could be an artifact of sequencing method, rather than actual deletion.

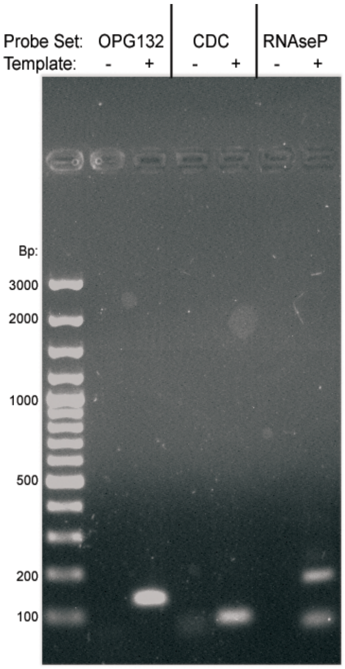

Supplementary Figure S5: 1% agarose gel showing the positive and negative control results for three qPCR probe sets. Ladder size markers are shown on the left.

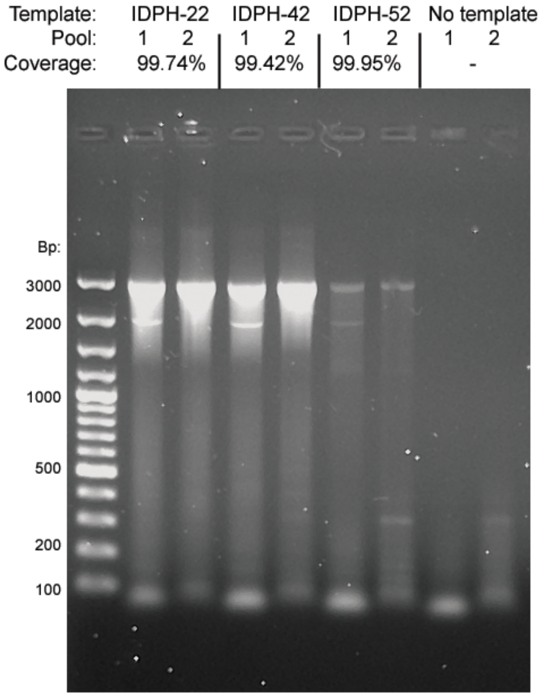

Supplementary Figure S6: 1% agarose gel showing three randomly-selected multiplex PCR samples, along with a negative control (no template). Final sequence coverage of each sample is also shown. Ladder size markers are shown on the left.

Table S1: NCBI Accession Numbers for Mpox Genomes.

MPXV-USA-IL-IDPH-001	OP536662	https://www.ncbi.nlm.nih.gov/nuccore/OP536662
MPXV-USA-IL-IDPH-002	OP536663	https://www.ncbi.nlm.nih.gov/nuccore/OP536663
MPXV-USA-IL-IDPH-003	OP536664	https://www.ncbi.nlm.nih.gov/nuccore/OP536664
MPXV-USA-IL-IDPH-004	OP536665	https://www.ncbi.nlm.nih.gov/nuccore/OP536665
MPXV-USA-IL-IDPH-005	OP536666	https://www.ncbi.nlm.nih.gov/nuccore/OP536666
MPXV-USA-IL-IDPH-006	OP536667	https://www.ncbi.nlm.nih.gov/nuccore/OP536667
MPXV-USA-IL-IDPH-007	OP536668	https://www.ncbi.nlm.nih.gov/nuccore/OP536668
MPXV-USA-IL-IDPH-008	OP536669	https://www.ncbi.nlm.nih.gov/nuccore/OP536669
MPXV-USA-IL-IDPH-009	OP536670	https://www.ncbi.nlm.nih.gov/nuccore/OP536670
MPXV-USA-IL-IDPH-010	OP536671	https://www.ncbi.nlm.nih.gov/nuccore/OP536671
MPXV-USA-IL-IDPH-011	OP536672	https://www.ncbi.nlm.nih.gov/nuccore/OP536672
MPXV-USA-IL-IDPH-012	OP536673	https://www.ncbi.nlm.nih.gov/nuccore/OP536673
MPXV-USA-IL-IDPH-013	OP536674	https://www.ncbi.nlm.nih.gov/nuccore/OP536674
MPXV-USA-IL-IDPH-014	OP536675	https://www.ncbi.nlm.nih.gov/nuccore/OP536675
MPXV-USA-IL-IDPH-015	OP536676	https://www.ncbi.nlm.nih.gov/nuccore/OP536676
MPXV-USA-IL-IDPH-016	OP536677	https://www.ncbi.nlm.nih.gov/nuccore/OP536677
MPXV-USA-IL-IDPH-017	OP536678	https://www.ncbi.nlm.nih.gov/nuccore/OP536678
MPXV-USA-IL-IDPH-018	OP536679	https://www.ncbi.nlm.nih.gov/nuccore/OP536679
MPXV-USA-IL-IDPH-019	OP536680	https://www.ncbi.nlm.nih.gov/nuccore/OP536680
MPXV-USA-IL-IDPH-020	OP536681	https://www.ncbi.nlm.nih.gov/nuccore/OP536681
MPXV-USA-IL-IDPH-021	OP536682	https://www.ncbi.nlm.nih.gov/nuccore/OP536682
MPXV-USA-IL-IDPH-022	OP536683	https://www.ncbi.nlm.nih.gov/nuccore/OP536683
MPXV-USA-IL-IDPH-023	OP536684	https://www.ncbi.nlm.nih.gov/nuccore/OP536684
MPXV-USA-IL-IDPH-024	OP536685	https://www.ncbi.nlm.nih.gov/nuccore/OP536685
MPXV-USA-IL-IDPH-025	OP536686	https://www.ncbi.nlm.nih.gov/nuccore/OP536686
MPXV-USA-IL-IDPH-026	OP536687	https://www.ncbi.nlm.nih.gov/nuccore/OP536687
MPXV-USA-IL-IDPH-027	OP536688	https://www.ncbi.nlm.nih.gov/nuccore/OP536688
MPXV-USA-IL-IDPH-028	OP536689	https://www.ncbi.nlm.nih.gov/nuccore/OP536689
MPXV-USA-IL-IDPH-029	OP536690	https://www.ncbi.nlm.nih.gov/nuccore/OP536690
MPXV-USA-IL-IDPH-030	OP536691	https://www.ncbi.nlm.nih.gov/nuccore/OP536691
MPXV-USA-IL-IDPH-031	OP536692	https://www.ncbi.nlm.nih.gov/nuccore/OP536692
MPXV-USA-IL-IDPH-032	OP536693	https://www.ncbi.nlm.nih.gov/nuccore/OP536693
MPXV-USA-IL-IDPH-033	OP536694	https://www.ncbi.nlm.nih.gov/nuccore/OP536694
MPXV-USA-IL-IDPH-034	OP536695	https://www.ncbi.nlm.nih.gov/nuccore/OP536695
MPXV-USA-IL-IDPH-035	OP536696	https://www.ncbi.nlm.nih.gov/nuccore/OP536696
MPXV-USA-IL-IDPH-036	OP536697	https://www.ncbi.nlm.nih.gov/nuccore/OP536697
MPXV-USA-IL-IDPH-037	OP536698	https://www.ncbi.nlm.nih.gov/nuccore/OP536698
MPXV-USA-IL-IDPH-038	OP536699	https://www.ncbi.nlm.nih.gov/nuccore/OP536699
MPXV-USA-IL-IDPH-039	OP536700	https://www.ncbi.nlm.nih.gov/nuccore/OP536700
MPXV-USA-IL-IDPH-040	OP536701	https://www.ncbi.nlm.nih.gov/nuccore/OP536701
MPXV-USA-IL-IDPH-041	OP536702	https://www.ncbi.nlm.nih.gov/nuccore/OP536702
MPXV-USA-IL-IDPH-042	OP536703	https://www.ncbi.nlm.nih.gov/nuccore/OP536703
MPXV-USA-IL-IDPH-043	OP536704	https://www.ncbi.nlm.nih.gov/nuccore/OP536704
MPXV-USA-IL-IDPH-044	OP536705	https://www.ncbi.nlm.nih.gov/nuccore/OP536705
MPXV-USA-IL-IDPH-045	OP536706	https://www.ncbi.nlm.nih.gov/nuccore/OP536706
MPXV-USA-IL-IDPH-046	OP536707	https://www.ncbi.nlm.nih.gov/nuccore/OP536707
MPXV-USA-IL-IDPH-047	OP536708	https://www.ncbi.nlm.nih.gov/nuccore/OP536708
MPXV-USA-IL-IDPH-048	OP536709	https://www.ncbi.nlm.nih.gov/nuccore/OP536709
MPXV-USA-IL-IDPH-049	OP536710	https://www.ncbi.nlm.nih.gov/nuccore/OP536710
MPXV-USA-IL-IDPH-050	OP536711	https://www.ncbi.nlm.nih.gov/nuccore/OP536711
MPXV-USA-IL-IDPH-051	OP536712	https://www.ncbi.nlm.nih.gov/nuccore/OP536712
MPXV-USA-IL-IDPH-052	OP536713	https://www.ncbi.nlm.nih.gov/nuccore/OP536713
MPXV-USA-IL-IDPH-053	OP536714	https://www.ncbi.nlm.nih.gov/nuccore/OP536714
MPXV-USA-IL-IDPH-054	OP536715	https://www.ncbi.nlm.nih.gov/nuccore/OP536715
MPXV-USA-IL-IDPH-055	OP536716	https://www.ncbi.nlm.nih.gov/nuccore/OP536716
MPXV-USA-IL-IDPH-056	OP536717	https://www.ncbi.nlm.nih.gov/nuccore/OP536717
MPXV-USA-IL-IDPH-057	OP536718	https://www.ncbi.nlm.nih.gov/nuccore/OP536718
MPXV-USA-IL-IDPH-058	OP536719	https://www.ncbi.nlm.nih.gov/nuccore/OP536719
MPXV-USA-IL-IDPH-059	OP536720	https://www.ncbi.nlm.nih.gov/nuccore/OP536720
MPXV-USA-IL-IDPH-060	OP536721	https://www.ncbi.nlm.nih.gov/nuccore/OP536721
MPXV-USA-IL-IDPH-061	OP536722	https://www.ncbi.nlm.nih.gov/nuccore/OP536722
MPXV-USA-IL-IDPH-062	OP536723	https://www.ncbi.nlm.nih.gov/nuccore/OP536723
MPXV-USA-IL-IDPH-063	OP536724	https://www.ncbi.nlm.nih.gov/nuccore/OP536724
MPXV-USA-IL-IDPH-064	OP536725	https://www.ncbi.nlm.nih.gov/nuccore/OP536725
MPXV-USA-IL-IDPH-065	OP536726	https://www.ncbi.nlm.nih.gov/nuccore/OP536726
MPXV-USA-IL-IDPH-066	OP536727	https://www.ncbi.nlm.nih.gov/nuccore/OP536727
MPXV-USA-IL-IDPH-067	OP536728	https://www.ncbi.nlm.nih.gov/nuccore/OP536728
MPXV-USA-IL-IDPH-068	OP536729	https://www.ncbi.nlm.nih.gov/nuccore/OP536729
MPXV-USA-IL-IDPH-069	OP536730	https://www.ncbi.nlm.nih.gov/nuccore/OP536730
MPXV-USA-IL-IDPH-070	OP536731	https://www.ncbi.nlm.nih.gov/nuccore/OP536731
MPXV-USA-IL-IDPH-071	OP536732	https://www.ncbi.nlm.nih.gov/nuccore/OP536732
MPXV-USA-IL-IDPH-072	OP536733	https://www.ncbi.nlm.nih.gov/nuccore/OP536733
MPXV-USA-IL-IDPH-073	OP536734	https://www.ncbi.nlm.nih.gov/nuccore/OP536734
MPXV-USA-IL-IDPH-074	OP536735	https://www.ncbi.nlm.nih.gov/nuccore/OP536735
MPXV-USA-IL-IDPH-075	OP536736	https://www.ncbi.nlm.nih.gov/nuccore/OP536736
MPXV-USA-IL-IDPH-076	OP536737	https://www.ncbi.nlm.nih.gov/nuccore/OP536737
MPXV-USA-IL-IDPH-077	OP536738	https://www.ncbi.nlm.nih.gov/nuccore/OP536738
MPXV-USA-IL-IDPH-078	OP536739	https://www.ncbi.nlm.nih.gov/nuccore/OP536739
MPXV-USA-IL-IDPH-079	OP536740	https://www.ncbi.nlm.nih.gov/nuccore/OP536740
MPXV-USA-IL-IDPH-080	OP536741	https://www.ncbi.nlm.nih.gov/nuccore/OP536741
MPXV-USA-IL-IDPH-081	OP536742	https://www.ncbi.nlm.nih.gov/nuccore/OP536742
MPXV-USA-IL-IDPH-082	OP536743	https://www.ncbi.nlm.nih.gov/nuccore/OP536743
MPXV-USA-IL-IDPH-083	OP536744	https://www.ncbi.nlm.nih.gov/nuccore/OP536744
MPXV-USA-IL-IDPH-084	OP536745	https://www.ncbi.nlm.nih.gov/nuccore/OP536745
