# Supplementary material for: Global mpox lineage discovery and rapid outbreak tracking with nanopore sequencing
Source: Virol J. 2023 May 6;20:90. doi: 10.1186/s12985-023-02059-2 (PMC10163848; doi:10.1186/s12985-023-02059-2)
Supplement: Supplementary file 2 — Additional file 2. WorkingProtocols. [file 12985_2023_2059_MOESM2_ESM.docx]

# Working Protocols

## Working Protocol 1 – DNA Extraction

### Introduction

The first step in the sequencing of Mpox virus genomes is extracting viral DNA from

positive Mpox samples. DNA is extracted from inactivated samples in lysis solution using

magnetic beads.

### Materials & Reagents

Note: All reagents were estimated for 96 samples. Omega Mag-Bind® Viral DNA/RNA 96 Kit is used for DNA extraction.

#### Reagents

- Viral Transport Media (VTM, 17.28 mL)
- Mag-Bind Bead Solution CNR (144 μL)
- 100 % Isopropanol (20.16 mL)
- Nuclease-free water (4.8 mL)

#### Materials

- 96-Well Deep-Well PCR Plate
- Bio-Rad Microseal B
- 70% Ethanol

#### Equipment

- BSL-2 Hood
- Full PPE
- Thermomixer
- 96-well magnetic platform

### Procedure

Caution**:** This is the step in the protocol when infection is possible. All steps are to be performed in

the BSL-2 biosafety cabinet. Full PPE should be worn during the entire extraction process. Samples should only be handled by authorized personnel.

#### Preparing Working Stock Lysis Buffer

1. If samples are not already inactivated by addition of Lysis Buffer, create a working stock
2. to supply 180 μL/sample.
3. Remove Carrier RNA from the -20°C freezer and allow to thaw.
4. Working stock lysis buffer is composed of 180 μL TNA Lysis Buffer and 0.75 μL carrier RNA.

***NOTE****: We developed a homemade lysis buffer as a substitute for the TNA lysis buffer,*

*which allows us to send pre-filled vials to testing facilities and send us back inactivated sample. The homemade lysis buffer performed similarly to TNA lysis buffer (data not shown). HM lysis buffer recipe: 100 mM sodium citrate (pH 6), 5 M guanidine isothiocyanate, 4% Triton X-100, 2 mM EDTA, 40 μg/mL purified (Torula) yeast tRNA.*

#### Inactivating Samples

1. Aliquot 150 μL of sample in VTM into each well of a 96-well deep-well plate.
2. Add 180 μL of Working Stock Lysis Buffer to each well with sample.
   1. If receiving frozen viral samples pre-mixed with homemade lysis buffer from testing facilities, allow samples to come to room temperature in biosafety cabinet and transfer to deep-well plate.
3. Seal the plate with a Bio-Rad Microseal B clear plate sealer.
4. Place the sealed plate on a thermomixer. Allow to mix at room temperature for 1 minute at 1100 RPM.

#### Binding DNA to Mag-Beads

1. Remove the Mag-Bind bead solution from 4°C storage and thoroughly vortex to resuspend beads.
2. Aliquot 211.5 μl of Mag-bind solution into each well of the plate, composed of 210 μL of 100 % isopropanol and 1.5 μL Mag-Bind beads. Mix solution by inversion and shaking of the tube before aliquoting. Note: Failing to mix can cause the beads to settle and unequally distribute, impacting purification yields.
3. Reseal the plate with Bio-Rad seal.
4. Place sealed-plate on thermomixer, and shake at RT for 10 minutes at 1100 RPM.

#### Separating and Washing Beads

1. After removing the plate from the thermomixer, place the plate in a 96-well magnetic platform designed to accommodate deep-well plates. Wait for the beads to be attracted to the magnet and cleared from the solution, approximately 10 minutes.
2. After solution is clear, carefully discard the supernatant, leaving beads untouched.
3. Wash the beads with VHB Buffer:
   1. Remove plate from magnetic platform and added 262.5 μl of VHB Buffer per well.
   2. Re-seal plate and place on thermomixer. Mix at RT for 1 minute at 1100 RPM.
   3. Place on magnetic platform for approximately 7 minutes to clear beads from solution.
   4. Carefully discard supernatant.
4. Wash the beads with SPR Wash Buffer:
   1. Remove plate from magnetic platform and added 262.5 μl of SPR Wash Buffer per well.
   2. Re-seal plate and place on thermomixer. Mix at RT for 1 minute at 1100 RPM.
   3. Place on magnetic platform for approximately 7 minutes to clear beads from solution.
   4. Carefully discard supernatant.
5. Wash the beads a second time with SPR Wash Buffer:
   1. Remove plate from magnetic platform and added 262.5 μl of SPR Wash Buffer per well.
   2. Re-seal plate and place on thermomixer. Mix at RT for 1 minute at 1100 RPM.
   3. Place on magnetic platform for approximately 7 minutes to clear beads from solution.
   4. Carefully discard supernatant.

#### Elution of DNA from Mag-Beads

1. After second wash, be sure to remove all remaining liquid from wells. A brief spin of the deep-well plate in a centrifuge to pool all liquids, followed by several more minutes on the magnetic platform to re-clear the magnetic beads is suggested.
2. Leave plate open inside biosafety cabinet for approximately 5 minutes to allow Mag-Bind particles to air dry. Do not over-dry Mag-Bind particles.
3. Remove deep-well plate from magnetic platform and add 50 μl of nuclease-free water. Alternately, Elution buffer (20 mM Tris, pH 7.2, 1 mM EDTA) can be used instead for more stable storage of extracted DNA samples.
4. Reseal plate and place on the thermomixer. Mix at room temperature for 10 minutes at 1100 RPM.
5. Place on magnetic platform for 10 minutes to clear beads from solution.
6. Transfer elution to new 0.3 mL qPCR plate.
7. Seal plate with a fresh seal and label appropriately.
8. Store at 4°C until ready for next step for same-day workflow. For long-term storage, store at -80°C.

## Working Protocol 2 – Viral Load estimation by qPCR

### Introduction

qPCR is used to confirm positive samples and determine which samples can result in full or nearly full genomes for the workflow. Any sample with a cycle threshold (*C*_t_) value exceeding a threshold *C*_t_ will not proceed through the workflow. This reduces cost and hands-on time. In addition to chances of genome retrieval, the *C*_t_ value provides insight on the viral load.

### Materials & Reagents

Note**:** All reagents & materials are estimated for 96 samples, scale as needed.

#### Reagents

- Nuclease-Free water (318 μL)
- 2X IDT PrimeTime (1060 μL)
- 10 μM N2 Forward Primer (106 μL)
- 10 μM N2 Reverse Primer (106 μL)
- 2.5 μM N2 Probe (106 μL)

#### Materials

- Bio-Rad 96 Well PCR Plate
- 96-Well Plate Seals

#### Equipment

- qPCR Instrument (Bio-Rad CFX-96)

### Procedure

#### Master Mix Assembly

Note**:** qPCR should be done on a clean bench to avoid any chance of contamination. Therefore,

decontaminate your bench with 70% ethanol before beginning.

1. Thaw 2X PrimeTime, 10 μM Forward Primer, 10 μM Reverse Primer, and 2.5 μM Probe
2. Gently flick, and spin down briefly before placing on ice.

Note**:** Avoid prolonged exposure of reference dye to light. Aluminum foil can be used to reduce exposure to light.

1. Prepare the master mix in a 2 mL microcentrifuge tube by combining the reagents in the following order, stopping after the 2.5 μM Probe.

**Reagents 1x 96x**

Nuclease-Free Water 3 μL 318 μL

2X PrimeTime 10 μL 1060 μL

10 μM Forward Primer 1 μL 106 μL

10 μM Reverse Primer 1 μL 106 μL

2.5 μM Probe 1 μL 106 μL

Sample DNA 4 μL -

Final Volume 20 μL 1696 μL

1. Retrieve the DNA plate from 4°C storage. Vortex and spin briefly. Place the plate directly on ice.
2. From the DNA plate, add 4 μL of cDNA to each well of the 96-Well qPCR plate, corresponding with their position on the plate layout.
3. Add 16 μL of the master mix to each well. Mix by pipetting up and down, gently.
4. Seal the PCR plate. Vortex then spin down the plate briefly.
5. Reseal the DNA extract plate store at 4°C or on ice until ready to use again.

#### qPCR Program

Run the following thermocycler conditions:

**Step Temp Time**

1 - Polymerase activation 95°C 3 min

2 - Denaturation 95°C 10 sec

3 - Annealing 55°C 30 sec

4 - Plate Read Read plate (Cy5)

Repeat steps 2-4 for 39 cycles.

This program will run for ~ 1 hour.

#### Data Retrieval

Set the baseline threshold at 200 and export the *C*_t_ values. Proceed with future steps with samples that have adequately low *C*_t_ values. (Typically *C*_t_ 32 or lower.)

## Working Protocol 3 – ARTIC-Style PCR Amplification

### Introduction

To have enough material to obtain sufficient reads during sequencing, DNA is exponentially amplified using PCR. This multiplex amplification protocol utilizes two pools of ARTIC-style primers which contains approximately 70 primers, each, to produce ~3000 bp amplicons.

### Materials & Reagents

Note**:** All reagents & materials are estimated for 96 samples, scale as needed. Primer scheme:

[https://github.com/gagnonlab/artic-mpxv/blob/main/Mpox_Sequencing_Primers.xlsx](https://github.com/gagnonlab/artic-mpxv/blob/main/Monkeypox_Sequencing_Primers.xlsx)

#### Reagents

- NEB 5X Q5 Reaction Buffer (Cat #: M0493L) (960 μL)
- NEB Q5 Hot-Start High-Fidelity Polymerase (Cat #: M0493L) (48 μL)
- 10 μM ARTIC-MPXV Primer Pool 1 Mix (IDT) (240 μL)
- 10 μM ARTIC-MPXV Primer Pool 2 Mix (IDT) (240 μL)
- NEB 10 mM dNTP Mix (Cat #: N0447L) (96 μL)

#### Materials

- 2x 96-Well PCR Plate

#### Equipment

- Thermocycler

### Procedure

#### Plate Setup

Create a new PCR layout that removes any sample with a C_t_ value greater than the chosen threshold. This layout we be utilized in future PCR steps.

#### Master Mix Assembly

PCR is extremely susceptible to contamination, work on a clean bench and wipe working areas with 70% ethanol. It is also highly advised to include a negative control (water only) beside the DNA samples and carry it forward to sequencing to determine the extent of contamination.

1. Thaw 5X Reaction Buffer, 10 mM dNTP mix, and Primer Pools 1 & 2. Mix at room temperature by vortexing, pulse centrifuge using a benchtop centrifuge and place on ice.
2. Dilute the 10 mM dNTP mix to 2.5 mM.
3. Gently flick the Q5 High-Fidelity Polymerase multiple times and immediately place on ice.
4. Dilute 100 μM primer pools in nuclease free water, to generate 10 μM primer stocks and place on ice.

Note: The MPXV primers are used at a final concentration of 12.5 nM per primer. Our initial sequencing observed a low yield in several amplicons. To overcome this, we spike in primers for these amplicons at 2x in the 10 μM primer set. These primers are for amplicon 43 in pool 1 and amplicons 18, 20, 38, 40, and 52 in pool 2.

1. Prepare the pool 1 and pool 2 master mixes:

**e (x96**

**Reagent 1x 96x)**

Nuclease-Free Water 11.25 μL 1,080 μL

5X Q5 Reaction Buffer 5 μL 480 μL

Pool 1 or 2 10 μM Primer Mix 2.5 μL 240 μL

2.5 mM dNTP Mix 2 μL 192 μL

Q5 High-Fidelity Polymerase 0.25 μL 24 μL

**Sub Total 21 μL 2016 μL**

1. Retrieve sealed PCR plate of extracted DNA samples. Spin down the plate briefly. Place on ice.
2. Transfer 4 μL of DNA for each sample that meets the Ct criteria to wells corresponding to the sample’s new plate position, for both Pool 1 and Pool 2 plates. The rest of the extracted DNA pcr plate can be stored at -20°C in case another reaction is desired.
3. Add 21 μL of Pool 1 Master Mix and Pool 2 Master Mix to the wells containing samples in plate 1 and plate 2, respectively.
4. Seal the PCR plate, making sure that each well is tightly sealed to prevent evaporation.
5. Vortex gently and spin the plate down.
6. Place plates in thermocyclers and run

#### Thermocycle Program

Step Cycle Step Temp Time Number of Cycles

1 Heat activation 98°C 0:30 1

2 Denaturation 94°C 0:16 21

3 Annealing / Extension 65-63°C 8:00 21

(Touchdown: -0.1 °C each cycle)

4 Denaturation 94°C 0:16 16

5 Annealing / Extension 63°C 8:00 16

6 Extension 72°C 4:00 1

7 Hold 4°C ∞

This program will run for ~ 5.5 hours.

1. After PCR is complete, it is recommended to confirm a successful reaction by running a selection of each of Pool 1 and Pool 2 samples, as well as Pools 1 and 2 of the negative control, on a 1% agarose gel. A lack of 3 kb bands in all lanes indicates something wrong with the PCR reaction, while bands in every well, including the negative control, could indicate major contamination that must be remedied before proceeding. Samples from DNA extracts with higher C_t_s (typically in the C_t_ 30 – 32 range) may produce weaker 3 kb bands. These can still be sequenced, but may require longer sequencing times.

## Working Protocol 4 - Bead Clean-up of PCR Products

### Introduction

The clean-up is performed by using AMPure XP SPRI beads to remove leftover PCR

contaminants (dNTPs, salts, primers, etc.) from the two previously performed PCR reactions.

Leftover contaminants may lead to poor DNA library end-prep and barcoding efficiency. The

advantage of using SPRI beads is their ability to size select DNA depending on the ratio of bead

to sample volume used. Expected recovery is 60-80%.

### Materials & Reagents

Note: All reagents & materials were estimated for 96 samples.

#### Reagents

- AMPure XP beads (4 mL)
- 100% Ethanol (40 mL)
- Omega Elution Buffer (EB) (2.9 mL)

#### Materials

- 96-well PCR plate
- 96-well magnetic separator
- Multichannel pipettes
- Plate Seal

### Procedure

You will begin this procedure with two 96-well plates containing the PCR product from the previously performed PCR reaction. The plates will be referred to as PCR pool 1 plate and PCR pool 2 plate.

Note: Amplicon clean up should be done on a clean bench to avoid any chance of contamination. Therefore, decontaminate your bench with 70% ethanol.

#### Pooling of PCR Products

CAUTION: Be very careful when pooling PCR products. Avoid cross-contaminating neighboring

wells with small droplets.

1. Spin down each PCR product plate of pool 1 and pool 2 at 2,000 RPM for 30 seconds. Remove seal on both plates.
2. Using an 8-channel P200 pipette, transfer all 25 μL from column 1 of PCR pool 2 plate to the corresponding wells of column 1 of the PCR pool 1 plate (Fig 1) and mix by pipetting up and down 3 times.

#### AMPure Bead Addition

1. Resuspend the stock AMPure XP Beads by vortexing for 20 seconds. The solution should be a homogenous brown color.
2. Using an electronic pipettor, remove 4 mL of AMPure XP beads and place it in a clean trough on ice. Place the stock beads back at 4°C.
3. Using an 8-channel P200 multichannel pipette set to 200 μL, mix the beads in the trough by pipetting up and down 5 times.
4. Immediately, use the multichannel pipette to transfer 37.5 μL of beads from the trough to each well of a column of the 96-well PCR plate. Repeat this with a fresh set of tips and each following column.
5. Between each transfer, mix the beads by pipetting up and down 2 times to ensure the beads do not settle.
6. Seal plate.
7. Incubate the plate at room temperature for 15 minutes to allow beads to bind to genomic DNA.

#### Purify the Pooled Reactions

CAUTION: Be careful not to touch the pelleted beads when removing the supernatant. Removal of beads will negatively impact yield.

1. After incubation, place 96-well plate on the magnet for 5 minutes. Check that all bead material has moved to the side of the well.
2. Carefully discard the supernatant without disturbing the beads.
3. Wash the beads with 200 μL of 80% ethanol.
4. Keep the plate on the magnet for 3 minutes.
5. Carefully discard the ethanol without disturbing the beads.
6. Repeat steps 3-5 (80% ethanol wash) for a second ethanol wash.
7. Centrifuge the 96-well plate at 2,000 RPM for 30 seconds to force any remaining ethanol wash to the bottom of the well.
8. Place the 96-well plate on the magnetic platform.
9. Use a P10 single channel pipette to aspirate any residual ethanol.
10. Let plate air-dry for 1-2 minutes to remove last traces of ethanol. Do not over-dry the beads.
11. Remove the plate from the magnetic platform and place onto the benchtop.
12. Elute DNA by adding 30 μL of Omega elution buffer to each of the well. Mix by pipetting up and down five times.
13. Cover the plate and incubate for 5 minutes at room temperature.
14. Place the plate on the magnetic platform for 5 minutes.
15. Carefully remove and retain 30 μL of the eluate containing the DNA library per well into a new 96-well PCR Plate. Retain the same plate layout.
16. Seal and label the new plate containing 30 μL of the eluate. Store at 4°C until ready for next step.

## Working Protocol 5 – Qubit Quantification

### Introduction

The Qubit dsDNA HS assay uses fluorescent dyes that bind specifically to dsDNA providing a more accurate quantification. This assay relies on a 2-point curve by reading the two standards for calibration every time measurements are taken. The assay is designed to quantitate 0.2–200 ng/μL of DNA.

### Materials & Reagents

#### Reagents

- Qubit dsDNA HS Buffer
- Qubit dsDNA HS Reagent (Dye)
- Qubit dsDNA HS Standard #1
- Qubit dsDNA HS Standard #2

#### Materials

- Qubit assay tubes
- Qubit 2.0 Fluorometer

### Procedure


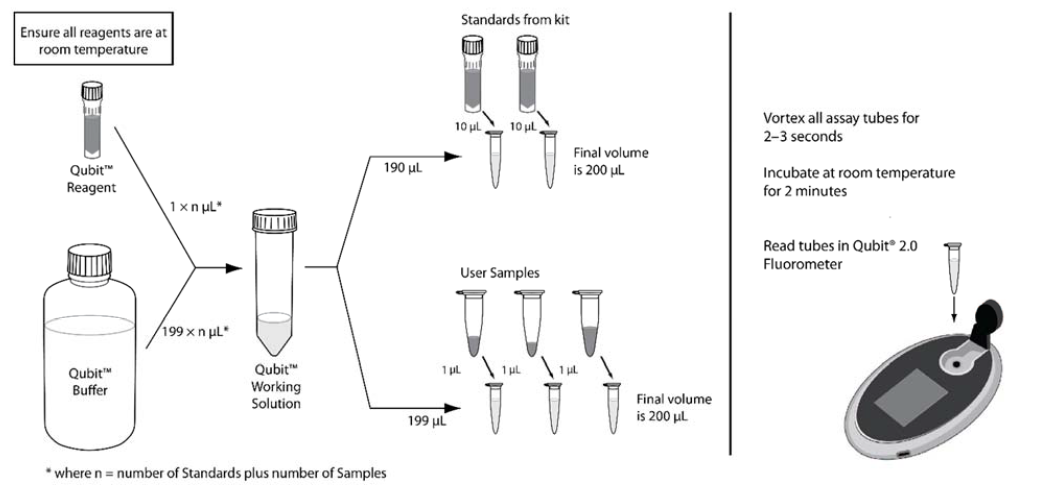


**Figure WP1. Workflow of Qubit quantification**

#### Qubit Reaction Preparation

1. Remove the Qubit standards and reagent (dye) from 4°C and let them equilibrate to room temperature.
2. Determine the total number of samples + two standards.
3. Set up two Qubit Assay tubes for the two standards and one tube for each sample to be quantified. Label the tube lids.
4. Make sufficient Qubit working solution (WS) in a single microcentrifuge tube for the total number of reactions (standards and samples), by combining 1 μL Qubit dsDNA reagent (dye) to 199 μL Qubit dsDNA buffer for each reaction. For example, for 96 samples, prepare enough working solution (WS) for a total of 100 reactions:

1X 100X

dsDNA Buffer 199 μL 1,990 μL

dsDNA Reagent 1 μL 100 μL

Total working solution 200 μL 2,000 μL

1. Mix the Qubit working solution (WS) by vortexing for ~4 seconds and briefly centrifuge.
2. Add 190 μL of Qubit working solution (WS) to each of the Qubit assay tubes used for standard 1 and standard 2.
3. Add 199 μL of Qubit working solution (WS) to each of the Qubit assay tubes used for samples to be quantified.
4. Add 10 μL of each Qubit standard to the appropriate tube, mix by vortexing for ~4 seconds and briefly centrifuge.
5. Add 1 μL of each DNA sample to the appropriate tube, mix by vortexing for ~4 seconds and briefly centrifuge.
6. Incubate tubes at room temperature for 2 minutes.

#### Qubit Quantification

1. On the Home screen of the Qubit Fluorometer, press **DNA**, then select **dsDNA High Sensitivity** as the assay type.
2. The “Read New Standards?” screen is displayed. Press **Yes.**
3. Insert Standard #1 into the Sample Chamber, close the lid and press **Read.**
4. Insert Standard #2, close the lid and press **Read.** Calibration of Qubit is now complete.
5. Choose **Sample** to go to the sample screen.
6. Insert a sample into the sample chamber, close the lid and press **Read.**
7. A result will display on the screen. Press **Calculate Stock Conc.**
8. Using the volume roller wheel, select **1 μL.**
9. Change the units in which the original sample concentration is displayed, select **ng/μL.**
10. Record the concentration value for the sample.
11. Repeat for all DNA samples by pressing **Read** once a new sample has been inserted.

## Working Protocol 6 – RAPID Barcoding

### Introduction

The barcoding reaction is done using the Nanopore Rapid Barcoding Kit 96 (SQK-RBK110.96). The barcodes are added via a transposase that also fragments the dsDNA. Due to the size of the Mpox genome, it is recommended to only sequence 20 genomes per flow cell. After barcoding, samples are pooled and cleaned up using beads.

### Materials & Reagents

#### Reagents

- Rapid Barcoding Plate

#### Materials

- 96-well PCR plate
- Plate seal
- Thermocycler

### Procedure

Note: Barcoding is done in a 10 μL reaction, utilizing 2.5 μL of Rapid Barcoding kit reagent per well. This allows for up to 7.5 μL of sample per reaction. Depending on number of samples, concentration of samples, and available time, it may be worth using different volumes of each sample in its individual reaction, making up remaining volume with nuclease-free water, in order to have each reaction have a similar nanogram amount of DNA per reaction. This will result in a more consistent sequencing experience.

1. Thaw the rapid barcode plate at room temperature.
2. Once thawed, centrifuge the rapid barcode plate briefly to ensure all reagent is pooled at the bottom of the well. Ensure that there is sufficient of each barcode being used per sample.
3. Transfer 7.5 μL of each sample (see above note) from Bead-Cleaned PCR Product plate (S4 protocol) to a new 96-well PCR plate. Save remainder of 30 μL in Bead-Cleaned PCR Plate by resealing and storing at -20°C.
4. Add 2.5 μL of the appropriate barcode reagent, 1-96, to each well of new PCR plate with sample in it. Mix by pipetting up and down 3 times.
5. Seal plate.
6. Gently vortex the plate to further mix samples. Centrifuge the plate at 2000 RPM for 1 minute to collect all liquid at the bottom of the well after vortexing.
7. Place the PCR plate in a thermocycler and run the following cycles:

#### Thermocycle Program

Step Cycle Step Temp Time

1 Barcoding 30°C 3:00

2 Inactivation 80°C 2:30

3 Hold 4°C ∞

This program will run for ~ 6 minutes.

## Working Protocol 7 – RAPID Sample Pool, Clean, & Adapter

### Introduction

After barcoding, all samples are pooled, cleaned, and then eluted into a single volume. This volume is quantified to determine the correct amount to load into a MinION flow cell. The RAPID adapter is then allowed to attach.

### Materials & Reagents

#### Reagents

- AMPure XP beads (4 mL)
- Elution Buffer
- 80% Ethanol

#### Materials

- Magnetic rack for Eppendorf tubes
- Qubit dsDNA HS Assay Kit
- Rotation mixer

### Procedure

#### Pooling and Bead Cleanup

1. Remove Qubit dsDNA HS reagents from 4°C and let them warm to room temperature.
2. Retrieve barcoding 96-well plate from S6 protocol.
3. Briefly centrifuge plate to pool all liquids at bottom of wells.
4. Unseal plate and pool all samples that are being loaded into a flow cell into an Eppendorf tube.

Note: Generally, approximately 20 samples can be easily loaded onto a single flow cell. If a full 96-well plate of samples has been prepared, divide them into multiple Eppendorf tubes of 19-20 samples each.

1. Resuspend AMPure XP beads by vortexing for 30 seconds.
2. Add 1x the volume of AMPure XP beads to the tube. Mix by pipetting up and down 5 times.
3. Incubate tube at room temperature for 10 minutes in a rotator mixer (Hula mixer).
4. Pulse-centrifuge the tube and place in magnetic rack for 5 minutes, or until solution is clear and beads have all collected on the side of the tube.
5. Carefully aspirate and discard the supernatant without disturbing the beads.
6. Add 500 μL of 80% ethanol while keeping the tube on the magnet.
7. Wait 3 minutes for the solution to clear and beads to collect on the side of the tube.
8. Remove ethanol and discard.
9. Repeat steps 10-12 for a second ethanol wash.
10. Briefly centrifuge tube to pool any remaining ethanol.
11. Place tube back on magnetic rack for an additional 3 minutes.
12. Use a P10 to aspirate any residual ethanol.
13. Let tube air-dry for 1-2 minutes to remove last traces of ethanol. Do not over-dry the beads.
14. Remove the tube from the magnetic rack and place onto the benchtop.
15. Elute DNA by adding 30 μL of Elution Buffer to each of the well. Mix by pipetting up and down five times.
16. Incubate at room temperature for 10 minutes.
17. Place the tube back on the magnetic rack for 5 minutes.
18. Transfer the 30 μL of DNA in elution buffer to a new Eppendorf tube. Keep on ice until ready to sequence.

#### Rapid Adapter Addition

1. As before, use the Qubit quantification protocol (S5) to quantify the ng/μL in the resulting elution tube. As the amount will most likely be above 200 ng/μL, it is recommended to first make a 1:10 dilution of the sample in water and use this for the quantification.
2. After quantification, calculate how many μL of sample needed to load 1500-2000 ng of dsDNA into the flow cell. Make up the remaining volume, up to 11 μL, in elution buffer.
3. Add 1 μL of Rapid Adapter F (RAP-F) to the 11 μL of barcoded DNA. Mix well by pipetting up and down 5 times.
4. Incubate at room temperature for at least 10 minutes to allow adapter to attach.

## Working Protocol 8 – MinION Loading and Running

### Introduction

The MinION flow cell is kept filled with a storage buffer to maintain product integrity. This must be flushed out and replaced. The pooled, barcoded DNA is then loaded and a sequencing run is started. Sequencing progress can be monitored in real-time using MinION software and RAMPART.

### Materials & Reagents

#### Reagents

- Loading Beads II – 25.5 μL
- Sequencing Buffer II – 37.5 μL
- Flush Buffer – 1 mL
- Flush Tether – 30 μL

#### Materials

- MinION
- Flow Cell

### Procedure

#### Prepare Priming Mix

1. Thaw the Sequencing Buffer II (SBII), Loading Beads II (LBII), Flush Tether (FLT) and Flush Buffer (FB). Place on ice once thawed.
2. Mix the SBII, FLT, and FB tubes by vortexing and then return to ice.
3. Open the MinION Sequencer lid (Figure 7) and remove the configuration cell.
4. Slide the flow cell into the sequence, under the clip. Press down gently on the flow cell to ensure correct thermal and electrical contacts.
5. Create Priming Mix by adding 30 μL of Flush Tether (FLT) into the new tube of Flush Buffer (FB). Label the FB tube to indicate FTL has been added. Mix the contents of the tube.


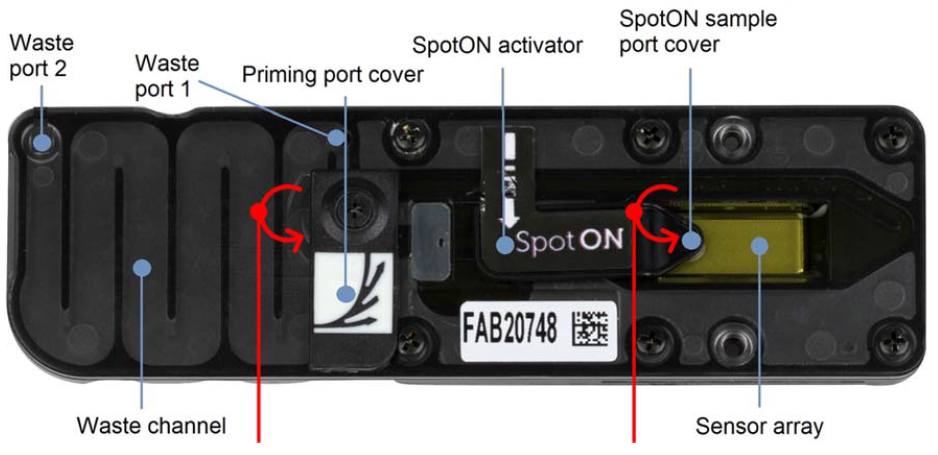


**Figure WP2. MinION flow cell**

#### Priming the Flow Cell

Caution: Throughout the following process it is essential that the sensor array remains submerged in buffer at all times. If an air bubble passes over any channels, those pores will be permanently damaged.

1. Open the priming port by sliding the cover clockwise so that the port is visible as shown in Figure S8.1.
2. Draw back a small volume of liquid from the priming port to remove any bubbles already present in the system:
   1. Insert a P1000 pipette tip into the priming port
   2. Adjust the P1000 volume upwards slowly until a small volume of liquid enters the pipette tip.
3. Slowly add 800 μL of Priming Mix to the priming port.
4. Close the priming port and allow flow cell to prime for five minutes. The Sequencing Mix can be prepared during this.

#### Prepare Sequencing Mix

Note: The Loading Beads II (LBII) tube contains a suspension of beads. These beads settle quickly. It is vital that they are mixed immediately before use by pipetting up and down.

1. In an Eppendorf tube, prepare the Sequencing Mix:

Reagent Volume

Sequencing Beads II 37.5 μL

Loading Beads II 25.5 μL

Barcoded DNA library 12 μL

Total 75 μL

1. Mix contents of tube and pulse-centrifuge to pool.
2. Store on ice until priming of flow cell is complete.

#### Loading Flow Cell

1. Open the flow cell priming port by sliding the cover clockwise.
2. Gently life the Spot-ON sample port cover to make the Spot-ON port accessible.
3. Slowly load another 200 μL of Priming Mix into the priming port. A droplet of buffer may rise from the Spot-ON port. Stop and wait for the droplet to recede before continuing to add the rest of the Priming Mix.
4. Resuspend the Sequencing Mix by gently pipetting up and down.
5. Add all 75 μL of Sequencing Mix to the flow cell via the Spot-ON sample port in a dropwise fashion. Each drop should flow into the port before adding another.
6. Close the Spot-ON port. Ensure the bung on the cover goes into the port hole.
7. Close the priming port.
8. Close the MinION lid.

#### Starting a Sequencing Run

1. Plug the MinION into the computer’s USB port.
2. Open MinKNOW, the sequencing software for the MinION.
3. Ensure the software recognizes the flow cell.
4. Go to the Connection Manager wind and click Start.
5. Name the experiment.
6. Click **Continue to Kit Selection**.
7. Select **SQK-RBK110.96** as the kit.
8. In the Run Options menu, leave at default settings.
9. In the Basecalling Menu, enable **Basecalling** and use the Options menu to configure the software for **Super-High Accuracy Basecalling**.
   1. Also enable **Barcoding**. Use the Options menu to set **Mid-read barcodes** to On. Set **Override minimum mid barcoding score** to 50.
10. In the Output menu, pick an output location and seq FAST5 and FASTQ Reads per File to 1000.
11. Click Start Run.

## Working Protocol 9 – Rampart Monitoring

### Introduction

Rampart (<https://artic.network/rampart>) is a useful program to supervise the progression of a MinION sequencing run. It uses the demultiplexed FASTQ read files that result from MinION base-calling and aligns them to a genomic template. This allows an estimation of coverage for each barcode being sequenced and is useful for determining how long to run the MinION sequencer.

### Materials

- Rampart can be installed using the directions found here: <https://artic-network.github.io/rampart/docs/installation.html>.
- Mpox genomic template and amplicon location files are needed for Rampart to function properly. These are available here: <https://github.com/gagnonlab/artic-mpxv>. After downloading, put the rampart directory in a known location.
- Location of MinION sequencing FASTQ files must be known.

### Procedure

1. Launch a console window from the Linux environment.
2. Activate the conda artic environment with the command:

conda activate artic-ncov2019

1. Launch rampart using the following syntax:

rampart --clearAnnotated --protocol [location of template file] --basecalledPath [location of FASTQ files]

For example:

rampart --clearAnnotated --protocol /home/gagnonlab/artic-mpxv/rampart/B1 --basecalledPath /var/lib/minknow/data/220815_mpxv/no_sample/20220815_1200_MN24287_FAP93592_f886cd67/fastq_pass/

1. Running this command will cause rampart to begin running in the console window. This produces a status window (Figure 8). Clicking on the **http://localhost:3000** link will open a browser window with detailed breakdowns of each barcode (Figure 9).
   1. Each barcode has its own panel which shows what percentage of the genome has 1x, 20x, 100x, and 200x coverage. It also shows how the reads match up along the length of the genomic, allowing analysis of low-amplifying amplicons.
   2. The **Report** button in the upper-right corner allows for numerical analysis of all barcodes at once: Average read length, coverage percentages, and percentage matching to the template genome.
   3. For best consensus-finding results, it is recommended to get at least 100x coverage of as much of the genome, for all sequences before terminating the sequencing run. If most genomes have good coverage but one or two barcodes are severely lagging, it is possible to load more of just those barcodes on the flow cell without the other barcodes to allow targeted read generation.


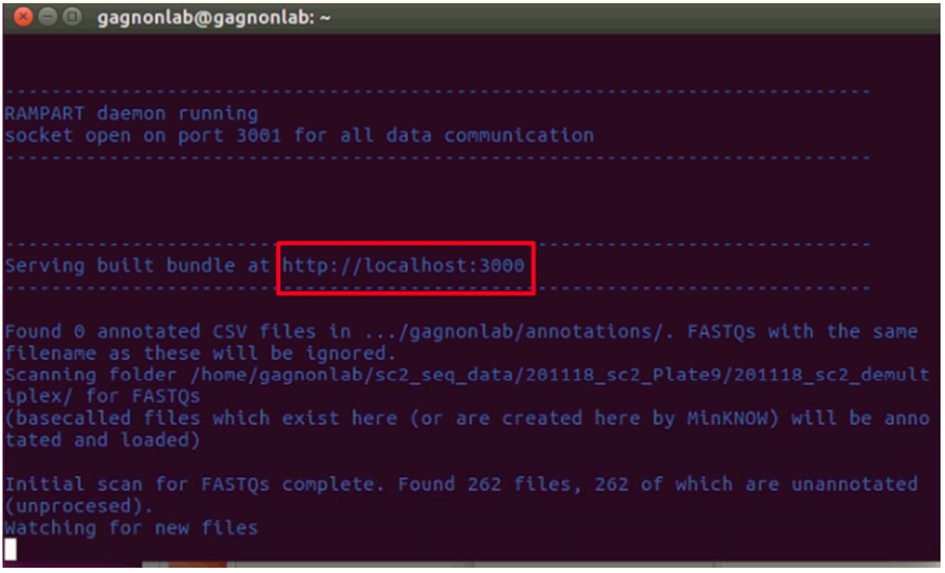


**Figure WP3. Rampart software.**


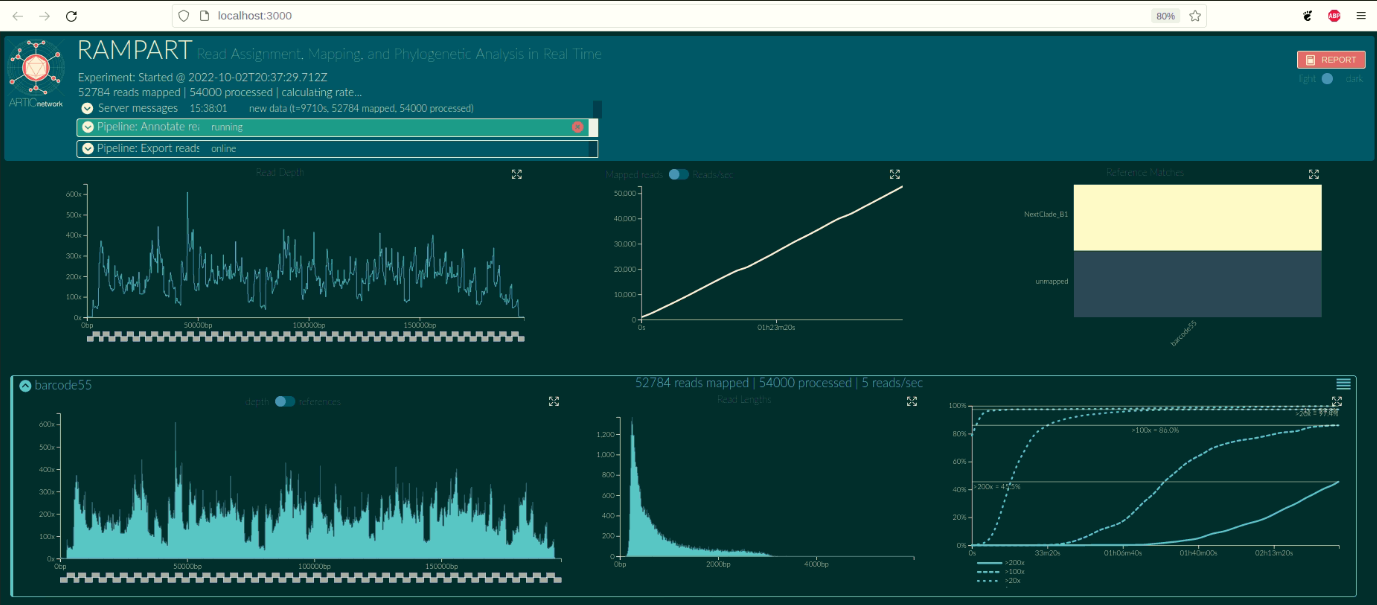


**Figure WP4. Rampart software output.**

## Working Protocol 10 – Consensus Sequence Calling

### Introduction

Once the MinION sequencing run is complete, the individual called reads need to be aligned against a template genome and compiled into a consensus sequence. The ARTIC network’s pipeline is useful for streamlining the consensus calling process.

### Materials

- The ARTIC pipeline installation instruction can be found here: <https://artic.readthedocs.io/en/latest/installation/>.
- This pipeline requires a Mpox-specific primer scheme and template genome in order to create a consensus sequence. A directory containing these files can be found at: <https://github.com/gagnonlab/artic-mpxv/tree/main/primer_schemes/B1/V1>.
- Two useful BASH files can be found at:

<https://github.com/gagnonlab/artic-mpxv/blob/main/concatBarcodes>

<https://github.com/gagnonlab/artic-mpxv/blob/main/consensusCall>

### Procedure

1. First, the FASTQ reads for each barcode need aggregated into one file that can be processed.
   1. Launch the ARTIC environment:

conda activate artic-ncov2019

- 1. An individual barcode can be aggregated using the following syntax:

artic guppyplex --directory [location of barcode FASTQ files] --skip-quality-check --output [name of file to aggregate FASTQ files into]

For example:

Artic guppyplex --directory /var/lib/minknow/data/220815_mpxv/no_sample/20220815_1200_MN24287_FAP93592_f886cd67/fastq_pass/barcode10/ --skip-quality-check --output barcode10.fastq

- 1. To aggregate a series of barcodes in a batch fashion, it is possible to use a BASH script, such as the **concatBarcodes** script linked in the Materials section. The format is:

./concatBarcodes FASTQ_DIR SAVE_DIR BARCODE_NUM_START BARCODE_NUM_END

For example:

./concatBarcodes /var/lib/minknow/data/220815_mpxv/no_sample/20220815_1200_MN24287_FAP93592_f886cd67/fastq_pass/ ./ 10 40

The above commands would aggregate the barcode FASTQ directories for barcodes 10 through 40 into files barcode10.fastq through barcode40.fastq.

1. After aggregation, the artic pipeline can be utilized to apply Nanopolish to the reads and deliver a consensus sequence file. Nanopolish requires FAST5 files and a sequencing summary in addition to the aggregated FASTQ reads in order to produce the consensus sequence.
   1. If not already in it, launch the ARTIC environment:

conda activate artic-ncov2019

- 1. An individual barcode can be consensus called with the artic pipeline command:

artic minion --normalise [number of reads for a given position on the genome to consider] --threads [number of CPU threads] --scheme-directory [location of mpox primer scheme] [name of Mpox primer scheme] --read-file [location of aggregated fastq file] [name of finished consensus fasta] --fast5-directory [location of FAST5 minION reads] --sequencing-summary [location of MinION sequencing run summary]

For example:

artic minion --normalise 200 --threads 12 --scheme-directory /home/gagnonlab/artic-mpxv/primer_schemes B1 barcode10 --read-file ./barcode10.fastq --fast5-directory /var/lib/minknow/data/220911_MPXV/no_sample/20220911_1348_MN31708_FAR89910_172c327d/fast5_pass/barcode10/ --sequencing-summary /var/lib/minknow/data/220911_MPXV/no_sample/20220911_1348_MN31708_FAR89910_172c327d/sequencing_summary_FAR89910_8b733801.txt

This will produce a file called barcode10.consensus.fasta in the directory the artic minion command was run from.

- 1. To derive a series of consensus sequences in a batch fashion, it is possible to use a BASH script, such as the **consensusCall** script linked in the Materials section. The format is:

./consensusCall BARCODE_DIR FAST5_DIR SUMMARY_PATH BARCODE_NUM_START BARCODE_NUM_END

For example:

./concatBarcodes ./ /var/lib/minknow/data/220911_MPXV/no_sample/20220911_1348_MN31708_FAR89910_172c327d/fast5_pass/ /var/lib/minknow/data/220911_MPXV/no_sample/20220911_1348_MN31708_FAR89910_172c327d/sequencing_summary_FAR89910_8b733801.txt 10 40

The above commands would use the aggregated FASTQ files named barcode10.fastq through barcode40.fastq, along with matching FAST5 files to derive consensus sequences, one after another, named barcode10.consensus.fasta, etc.

## Working Protocol 11 – Visualizing Consensus Sequences

### Introduction

When generating consensus sequences, it is important to check the result to ensure that nothing has gone wrong in the sequencing or consensus calling. NextClade, a website run by the NextStrain community, was originally designed for analyzing SARS-CoV-2 genomic sequences, but has since expanded to include Mpox and Influenza strains as well.

If needed, there is also an offline command-line interface version of NextClade, for processing a large number of samples.

### Procedure

1. Navigate to <https://clades.nextstrain.org/> to launch the NextClade web interface.
2. Select pathogen of interest. For current 2022 outbreak mpox samples, select **Human Monkeypox Lineage B.1**.
3. Supply Nextclade with FASTA-format sequences. This can either be done by dragging-and-dropping the file into the website, or by copying and pasting the FASTA as text.
4. Click the **Run** button to have the website analyze the samples.


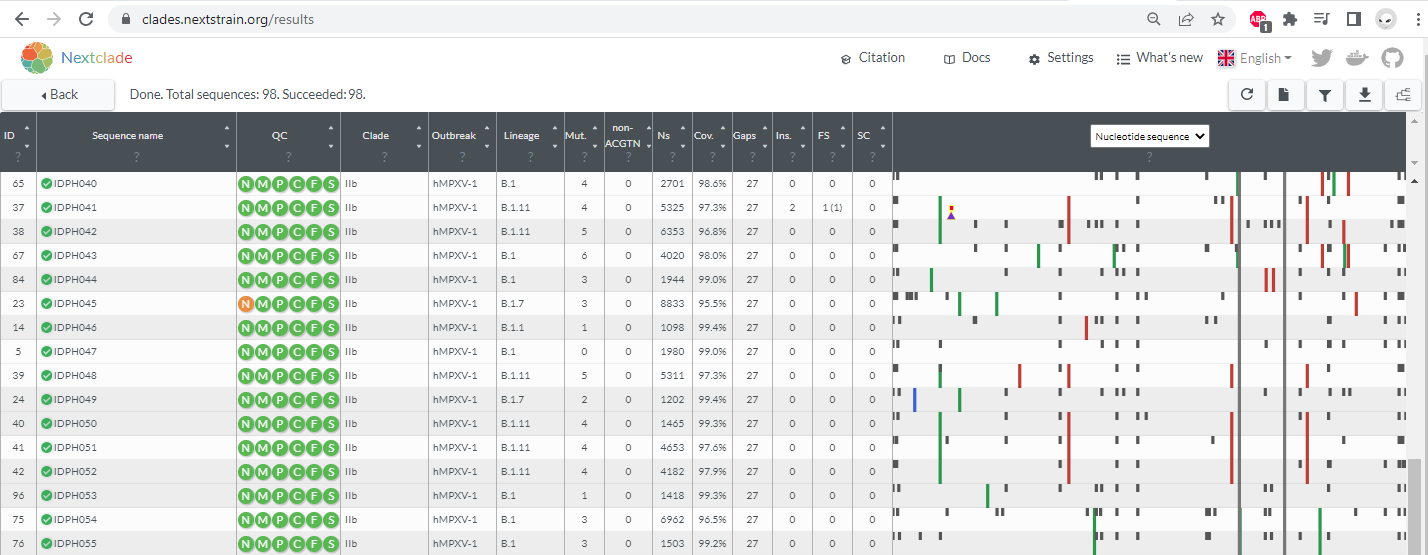


**Figure WP5. NextClade software output.**

Each column (Figure 10) provides different information about your sequence:

- Sequence name – name provided for sequence in FASTA file
- QC – General indications of quality of sequence. In general, green and yellow are acceptable, red is unacceptable. Hovering the mouse over each circle gives more details.
  - N circle – measurement of missing data. Based on the number of Ns in the sequence.
  - M circle – measurement of mixed sites. Will be flagged if more than 10 nucleotides have mixed states (such as R or Y), as this may be indicative of contamination.
  - P circle – measurement of private mutations. Based on the number of mutations present compared to the template sequence.
  - C circle – measurement of mutation clusters. Based on the clustering of mutations. If more than 6 mutations are within a 100 base range, the sequence will be flagged.
  - F circle – measurement of frame shifts.
  - S circle – measurement of unusual stop codons.
- Lineage – What Nextstrain lineage the software determines the sequence to be. This label is reliable with good quality reads, but may be unreliable with too many missing nucleotides.
- Mut. – Number of mutations in the sequence compared to template genome. Hovering the mouse over a specific sequence’s mutation column will generate a popup window with a list of nucleotide and amino-acid mutations.
- Non-ACGTN – Number of ambiguous, non-N nucleotides in the sequence. Hovering the mouse over a specific sequence’s Non-ACGTN column will generate a list.
- Ns – Number of missing nucleotides in the sequence. Hovering the mouse over a specific sequence’s Ns column will generate a list.
- Gaps – Number of intentional gaps in the sequence (-). Produced by deletions in the nucleotide code. Hovering the mouse over a specific sequence’s Gaps column will generate a list.
- Ins. – Number of insertions in the sequence. Hovering the mouse of a specific sequence’s Ins. Column will generate a list.
- FS – Number of frame shifts in the sequence. Hovering the mouse of a specific sequence’s FS Column will generate a list.
- SC – Number of premature stop codons in the sequence. Hovering the mouse of a specific sequence’s SC Column will generate a list.
- Sequence View – Color-coded visual representation of the mutations, gaps, and Ns in the sequence, as compared to template genome. Red, Mutation to A. Blue, Mutation to C. Yellow, mutation to G. Green, mutation to T. Dark grey, N. Light grey, gap. A visual representation of Mpox genes is provided below the sequences for comparison. Hovering the mouse over a specific color-coded bar will provide additional information about it.

In addition, there are several useful tool buttons at the top of the page:

- Filter – Funnel icon. Filter your sequence list based on various criteria, including a mutation at a specific nucleotide or amino-acid, the quality of the sequence, or the lineage it is categorized as.
- Download – Down pointing arrow icon. Allows the generation of a spreadsheet with all details of the analysis for later browsing.
- Show Tree – Phylogenetic tree icon. Places sequences on a phylogenetic tree for comparison with other sequences from the public sources.
- Settings – Gear icon. Adjust settings for each of the four quality control determinations.

Using these criteria, poor-quality sequences can be identified and removed from your FASTA file.
